# Sustained chemogenetic activation of locus coeruleus norepinephrine neurons promotes dopaminergic neuron survival in synucleinopathy

**DOI:** 10.1371/journal.pone.0263074

**Published:** 2022-03-22

**Authors:** Predrag Jovanovic, Yidan Wang, Jean-Philippe Vit, Edward Novinbakht, Nancy Morones, Elliot Hogg, Michele Tagliati, Celine E. Riera

**Affiliations:** 1 Center for Neural Science and Medicine, Biomedical Sciences Department and Board of Governors Regenerative Medicine Institute, Cedars-Sinai Medical Center, Los Angeles, CA, United States of America; 2 Department of Neurosurgery, Cedars-Sinai Medical Center, Movement Disorder Program, Los Angeles, CA, United States of America; 3 Department of Neurology, Cedars-Sinai Medical Center, Movement Disorder Program, Los Angeles, CA, United States of America; 4 David Geffen School of Medicine, University of California, Los Angeles, CA, United States of America; Thomas Jefferson University, UNITED STATES

## Abstract

Dopaminergic neuron degeneration in the midbrain plays a pivotal role in motor symptoms associated with Parkinson’s disease. However, non-motor symptoms of Parkinson’s disease and post-mortem histopathology confirm dysfunction in other brain areas, including the locus coeruleus and its associated neurotransmitter norepinephrine. Here, we investigate the role of central norepinephrine-producing neurons in Parkinson’s disease by chronically stimulating catecholaminergic neurons in the locus coeruleus using chemogenetic manipulation. We show that norepinephrine neurons send complex axonal projections to the dopaminergic neurons in the substantia nigra, confirming physical communication between these regions. Furthermore, we demonstrate that increased activity of norepinephrine neurons is protective against dopaminergic neuronal depletion in human α-syn A53T missense mutation over-expressing mice and prevents motor dysfunction in these mice. Remarkably, elevated norepinephrine neurons action fails to alleviate α-synuclein aggregation and microgliosis in the substantia nigra suggesting the presence of an alternate neuroprotective mechanism. The beneficial effects of high norepinephrine neuron activity might be attributed to the action of norepinephrine on dopaminergic neurons, as recombinant norepinephrine treatment increased primary dopaminergic neuron cultures survival and neurite sprouting. Collectively, our results suggest a neuroprotective mechanism where noradrenergic neurons activity preserves the integrity of dopaminergic neurons, which prevents synucleinopathy-dependent loss of these cells.

## Introduction

The demise of catecholaminergic neurons, characterized by Lewy pathology and loss of dopaminergic (DA) neurons in the substantia nigra pars compacta (SNc), is a dominant feature of Parkinson’s disease (PD). DA neuronal death is linked to motor impairments including bradykinesia, tremor, muscular rigidity and postural instability [1, 2]. Large protein-rich intracellular inclusions known as Lewy bodies (LB) or Lewy neurites (LN) are associated with chronic inflammation [3, 4]. Alpha-synuclein (α-syn) is the main component of LB and recognized as the major protein underlying PD pathogenesis as documented in genetic studies [5] and animal models overexpressing human α-syn [6, 7]. α-syn, encoded by the SNCA gene, is abundant in the central nervous system (CNS) where it localizes to presynaptic terminals and participate in vesicular trafficking [8, 9].

While the most recognized clinical features of PD are its motor symptoms, PD is a multifaceted disease with an abundance of non-motor symptoms including anxiety, depression, rapid eye movement (REM) sleep behavioral disorder, constipation and cognitive decline. Importantly, these non-motor symptoms often occur years or even decades prior to the onset of motor dysfunction [10–13]. Lewy body pathology and degeneration of catecholamine cell groups in the midbrain, pons, and medulla are frequently observed and may precede SNc degeneration [14]. In particular, significant cell death in the locus coeruleus complex (LC) [15], located in the pons, and the presence of α-syn aggregation in that region are core features of PD [1, 3, 4, 15]. According to Braak’s theory, the LC is involved with early stage spreading of α-syn to interconnected nuclei, followed by SNc and amygdala regions [16]. This observation has been confirmed by histopathological studies demonstrating early and extensive LC degeneration [14].

Experimental studies suggest that the central norepinephrine (NE) system might significantly influence the onset and disease progression in the midbrain. Importantly, increasing synaptic NE by genetic deletion or pharmacological blockade of the NE transporter (NET) confers resistance to depletion of DA neurons in models of MPTP toxicity and progressive dopaminergic neurodegeneration [17–19]. In line with these observations, mice mutant for dopamine β-hydroxylase (DBH), unable to synthesize NE, develop motor deficits [20]. NE is supplied to the whole brain mainly by constitutively active tyrosine hydroxylase (TH) neurons in the LC, through extensively ramified axons which release both synaptic and extra-synaptic NE [21, 22]. It is assumed that LC neurons produce more than half of all brain NE [23, 24]. The cellular responses to exogenous NE depends on the adrenergic receptors (ARs) expressed within each cell type, as these G-protein coupled receptors present Gq-, Gi/o-, or Gs-modulatory properties [25].

Because of the temporal relationship between LC and SNc pathology, variations in LC-NE activity could potentiate SNc neuron vulnerability to α-syn toxicity. Rodent and primate research demonstrates that lesions in the LC accelerate 6-hydroxydopamine (6-OHDA)- and 1-methyl-4-phenyl-1,2,3,6-tetrahydropyridine (MPTP) -mediated SNc degeneration [20, 26–28]. Whether DA neuron survival in the face of α-syn aggregation can be enhanced by temporal stimulation of LC-NE neurons remains unknown. Here, we show that select stimulation of these neurons, over a period of six weeks, has a neuroprotective action on DA neurons in the striatum and consequently ameliorates motor coordination in a mouse model of pathogenic α-syn aggregation. We also demonstrate that ectopic treatment of NE on primary DA neurons expressing pathogenic α-syn increases survival and neurite sprouting in culture. These results highlight a fundamental function for LC neurons in PD pathophysiology and provide key insight into the role of the noradrenergic system in the neuromodulation of DA neuron survival.

## Materials and methods

### Experimental design

All procedures were approved by the Animal Care and Use Committee of Cedars Sinai Medical Center. Human Synuclein mutation A53T (Jax strain 006823) and rat TH promoter directing expression of Cre recombinase to catecholaminergic cells (Jax strain 008601) mice were obtained from Jackson Laboratories and bred to generate heterozygote male and female experimental mice. Transgenic A53T mice express the human A53T mutant α-syn protein under the control of the prion promoter (SNCA mice), resulting in approximately six times the level of endogenous mouse α-syn [29]. Mice were maintained on a 12 h: 12 h light:dark cycle, fed normal chow (PicoLab Rodent 20 5053*, LabDiet).

### Stereotaxic surgery

Anesthesia was induced using isoflurane (induction, 5%; maintenance, 1–2%). Mice were placed on the stereotactic frame (Kopf Instruments). A small hole was drilled into the skull over the target site. 8-month-old TH-Cre^+^;SNCA^+^ and control littermates were injected with adeno-associated virus encoding double floxed Gq-coupled hM3D DREADD fused with mCherry under the control of human synapsin promoter (Addgene, 44361) bilaterally (300 nl on each side) at a rate of 2 nl sec-1 into the target site, using a microliter syringe (Hamilton) and UMP3 pump (WPI) mounted directly on the stereotactic frame. After the injection, the needle of the syringe was left on the skull for 10 min to reduce backflow and then withdrawn slowly. The coordinates for the LC were (relative to bregma) anteroposterior −5.4 mm, lateral +/- 0.8 mm and dorsoventral -4.0 mm. Carprofen (0.08 ml kg^−1^ body weight) and buprenorphine (0.1 mg kg^−1^ body weight) were administered subcutaneously to all mice before the surgical procedure. Carprofen is neuroprotective and induces cell proliferation and gliogenesis after traumatic brain injury [30]. Animals were recovered on a heating pad until normal behavior resumed.

### Treatment

Clozapine-N-Oxide (CNO) was delivered by osmotic minipumps (Alztec, model 2006) at a constant rate of 0.15μl/hr for 50 days. Implantation of minipumps was performed one month after intracranial virus injection. Pumps were filled with either saline (0.9% sodium chloride) or CNO (0.75 mg kg−1 body weight per day). To implant the pumps, mice were anesthetized using isoflurane (induction, 5%; maintenance, 1–2%) and an incision of the appropriate length was made in the dorsal subcutaneous skin to create a subcutaneous dead space. The filled pump was placed into the dead space but not directly beneath the incision. Carprofen (0.08 ml kg−1 body weight) was administered subcutaneously before the procedure.

### Indirect calorimetry, physical activity, food intake and body composition

Indirect calorimetric studies were conducted in an automated home cage eight-chamber phenotyping system (Phenomaster, TSE). Body composition was assessed by EchoMRI. Mice were acclimatized in the chambers for at least 24 hours. Food and water were provided ad libitum in the appropriate devices and measured by the built-in automated instruments. Locomotor activity and parameters of indirect calorimetry were measured for at least the following 72 hours.

### Open field test

Spontaneous locomotor activity was measured using the Photobeam Activity System (San Diego Instruments), which consists of a 40cm-by-40cm square chamber with 40cm high clear Plexiglass walls and virtually divided into center (30cm-by-30cm) and perimeter (5cm along each wall). Two rings of 16 photobeams and optical sensors surrounded the chamber and beam breaks captured horizontal ambulatory activity and vertical rearing behavior of the mouse. Each mouse was individually placed in the center of the chamber and left undisturbed to freely explore the environment for a 60-min period. Number of rearing, total activity, percentage of center activity were recorded. Distance, average speed, resting time and trajectory of each mouse were computed using PAS Reporter and PAS PathView softwares.

### Rotarod

The rotarod test was used to measure motor function and coordination. Each mouse was placed on a rotating rod elevated 18 inches above a soft surface and set at a constant speed of 3 rpm for 30s, then immediately followed by a gradual acceleration from 3 rpm to 30 rpm over a 3-min period. For 3 consecutive days, the latency to fall off the rod and the distance traveled when the fall occurred were averaged across 3 trials separated with 30-min inter-trial resting periods where the mouse was placed back into its home cage.

### Fasting glucose measurement

After a 16 hour fast, blood glucose concentration was measured using an AlphaTRAK 2 glucometer (Zoetis) via tail bleeding.

### Insulin sensitivity test

5 hours fasted mice were injected with (1u/kg weight) human insulin (Humalog, Lilly) and glucose levels were measured over the course of 120 minutes.

### Tissue preparation

Mice were anesthetized with isoflurane and perfused transcardially with ice cold 0.1M potassium phosphate-buffered saline (PBS, pH 7.4) followed by 4% paraformaldehyde (PFA). Brains were removed, stored in the same fixatives, and transferred into 20% sucrose at 4°C overnight. The brains were embedded in optimal cutting temperature (OCT) compound (Tissue-Tek) and cut into 40 microns coronal sections at the level of the substantia nigra (-2.67 mm to -2.97 mm from bregma and locus coeruleus −5.27 mm to -5.47 mm) on a Leica cryostat.

### Cryo-section immunostaining

Brain sections were incubated in PBS buffered blocking solution containing 2% normal donkey serum and 0.2% Triton X-100 for 1 hour at room temperature, followed by incubation overnight at 4°C in blocking solution containing primary antibodies: anti-alpha-synuclein (1:500, ab80627, abcam); anti-iba1 (1:500, 019–19741, Wako); anti-mCherry (1:1000, PA5-34974, Thermo Fisher); anti-TH (1:500, ab1542, ab152, Sigma-Aldrich), Nissl neurotrace 640/660 (1:300, Thermo Fisher N21483). The sections were washed three times in PBS and then incubated in one of the following secondary antibodies for 1 h in the dark at room temperature: donkey anti-rabbit Alexa Fluor® 488(1:500, A21206, Thermo Fisher); donkey anti-mouse Alexa Fluor® 488 (1:500, A21202, Thermo Fisher); donkey anti-sheep Alexa Fluor® 488 (1:500, A11015, Thermo Fisher); Cy5 ® (1:500, ab6564, Abcam). Sections were washed three times in PBS, mounted onto slides, coverslipped with ProLong® Gold Antifade Reagent with DAPI (8961S, Cell Signaling). For detection of the phosphorylated form of α-syn at serine 129 (pSer129) sections were incubated in formic acid for 5 min before proteinase K digestion (Enzo Life Sciences, Farmingdale, NY) (1:100 for 15 min at 37°C water followed by 5 min at room temperature) prior to incubation with primary antibody pSer129 α-syn (1:500, ab51253, Abcam).

### Primary dopamine neurons cultures

Ventral midbrain region containing SNc and VTA were dissected from C57BL/6J neonates of both sexes. Meninges were removed, and brain tissues pieces were dissociated for 15 minutes in a papain dissociation, washed three times with Hank’s Balanced salt solution (Gibco) containing 10mM HEPES and 20mM glucose, triturated and plated on poly-L-lysine and laminin coated chamber slides. Neurons were plated in Hams F12/DMEM + Glutamax, B27 without antioxidant, 1% penicillin/streptomycin, 10% fetal bovine serum, and uridine and 5-fluoro-s-deoxyurine to inhibit glial proliferation. Cells were transinfected with AAV1/2-CMV-CBA-human-A53T-alpha-synuclein-WPRE-BGH-polyA (Genedetect, GD1001-RV) or AAV1/2-CMV/CBA-GFP-WPRE-BGH-polyA (Genedetect, GD1004-RV) 24 h post-isolation at a multiplicity of infection (MOI) of 3000. Media containing AAV vectors were removed after 72 h. NE (Sigma-Aldrich, A7256) or glial gell-line derived neurotrophic factors (GDNF) (Sigma-Aldrich, SRP3200) were added to cell media every 24 h. Half media change was done every 48–72 h. Cells were fixed with 4% paraformaldehyde for immunofluorescence staining at 4-to 8-days post-isolation.

### Visualization and analysis

Fluorescent sections were imaged with either a BZ-X700 Keyence and Zeiss Axio observer microscope 7 with apotome 2.0. To count DA neurons in SNc and LC, three sections containing both hemisheres for each region of interest (ROI) were counted and the average number of neurons calculated. The ROI was matched for location across genotypes, by confirming with Paxinos and Franklin’s mouse brain atlas and QuickNII [31]. Coordinates of ROI were as follows: SNcA/P (-2.79mm to -3.39mm) and LC A/P (−5.27 mm to -5.47 mm). 10X images were converted to 8-bit grayscale and adjusted for brightness and contrast to best visualize all TH positive neurons. The plugin Cell Counter was used to count the number of neurons expressing TH immunoreactivity manually by 3 blinded experimenters. The exact field of view (FOV) was estimated using BZ-X700 Keyence Analyzer tool. The total number of TH neurons/mm^3^ for each mouse was calculated as *N(neurons/mm*^*3*^*) = [Nneurons/FOV (mm*^*2*^*) x section thickness (mm)]*. Intact cells percentage were calculated by number of intact cell bodies over total cell numbers. Total area of α-syn aggregation and particle sizes were quantified using an automated threshold-based algorithm by BZ-analyzer Hybrid Cell Count function under 40X images. For SNc, 40X pictures of medial and lateral part of SNc were taken on both sides. Total area of iba1 positive cells and soma size were quantified using the same algorithm by BZ-analyzer Hybrid Cell Count function. A skeleton analysis method was used to quantify microglia branch length and branching complexity as previously described using ImageJ [32]. DA neurons growth was analyzed using NeuronJ plugin in ImageJ. 20X or 40X images were converted to 8-bit grayscale and adjusted brightness and contrast. Neurite growth was traced, and number of roots were counted manually. Total neurite length, mean neurite length, and number of extremities were measured by ImageJ. Number of branch points were calculated by subtracting number of roots from extremities.

### Statistical analysis

GraphPad Prism 8 was used for statistical analysis. Data are expressed as mean ± SEM. p Values were calculated using one-way or two-way ANOVA with post hoc Tukey tests as appropriate. P values less than 0.05 were considered significant.

## Results

### Stimulation of noradrenergic neurons slows progression of PD-like motor symptoms in mice

To explore the possible relationship between LC-NE activity and α-syn aggregation in PD, we evaluated the consequence of chronic stimulation of TH-expressing neurons in a mouse model of α-syn aggregation. Mice over-expressing human α-syn A53T missense mutation (referred as SNCA mice) are a widely used transgenic model of pathological α-syn and develop adult-onset neurodegeneration between 9 and 16 months of age, characterized by sustained abnormal posturing and reduced spontaneous motor activity [29, 33]. This mouse model possesses face validity as it is characterized by an accumulation of α-syn within neuronal cell bodies and neurites, with affected brain regions displaying detergent-insoluble α-syn and α-syn aggregates. This model therefore exhibits key features of PD, including α-syn inclusions, accumulation of ubiquitin, neurofilaments and motor phenotypes.

**Fig 1:**
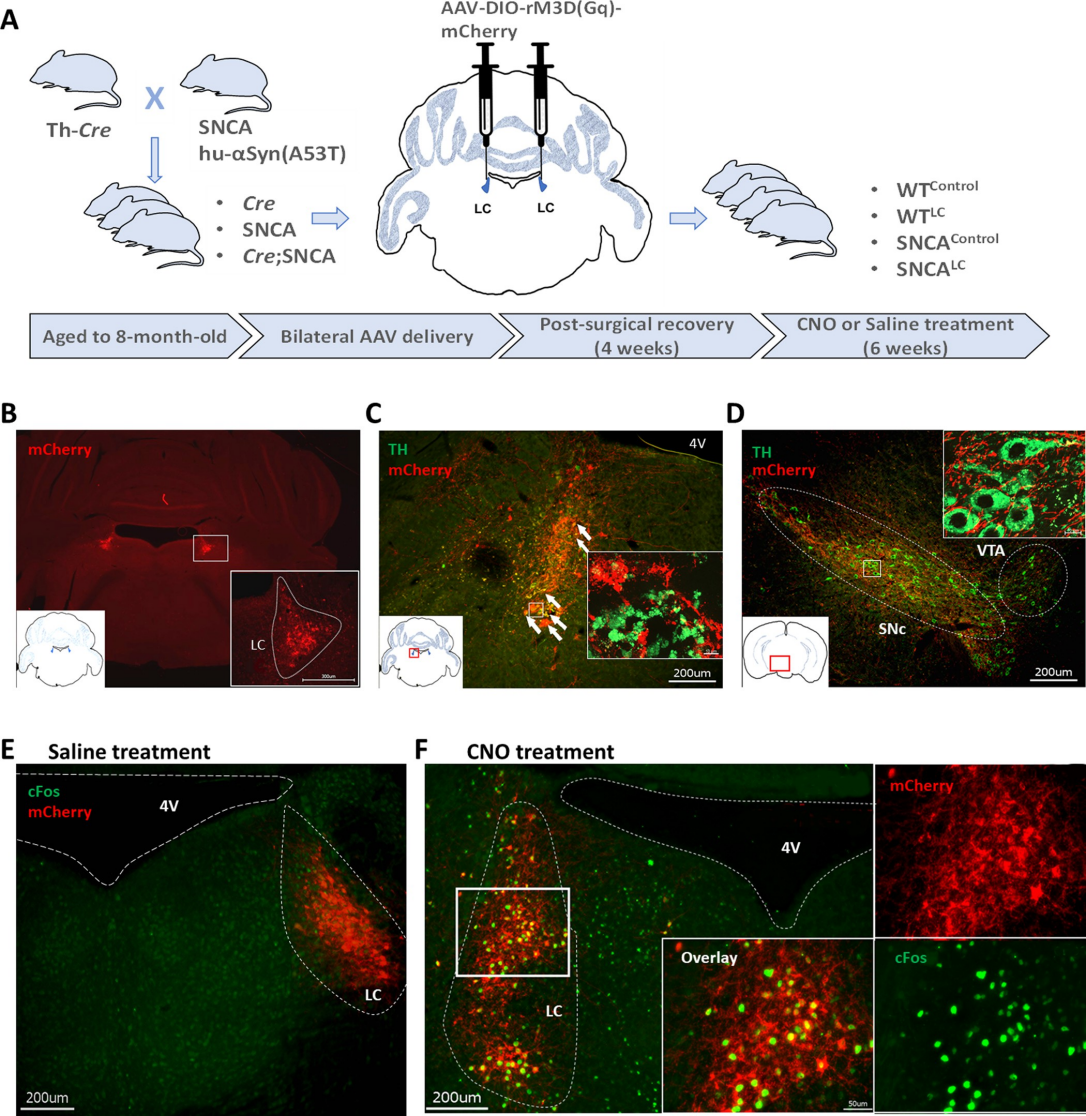
Chronic stimulation of LC-NE neurons using hM3D-DREADD. A- Experimental approach to chronically activate LC-NE neurons. B- Coronal section showing mCherry labeled neurons (red) at the injection sites (locus coeruleus), scale bar 300μm. C- Representative image showing colocalization of mCherry (red) and TH positive cells (green) in locus coeruleus, scale bar 200 μm. D- Representative image showing LC-NE projections to substantia nigra, mCherry positive fibers (red), TH positive cells (green), scale bar 200 μm. E, F-Activation of TH neurons in LC, transduced with AAV containing double floxed Gq-coupled hM3D DREADD fused with mCherry under the control of human synapsin promoter, upon injection of saline or CNO, respectively, mCherry (red) and c-Fos positive cells (green) in locus coeruleus, scale bar 50 μm and 200 μm.

LC-NE neurons form a densely packed population of cells (roughly 1600 cells per LC in mice) which produce NE [34, 35]. We used Designer Receptors Exclusively Activated by Designer Drugs termed DREADDS [36] to chemogenetically activate LC-NE neurons of SNCA animals. We delivered a *Cre*-dependent AAV expressing excitatory hM3Dq and carrying a mCherry reporter gene, into the bilateral LC of eight-month-old hemizygous mice of littermate animals of the following genotypes: *Th-Cre*^*+/-*^*;SNCA*^*-/-*^, *Th-Cre*^*-/-*^*;SNCA*^*+/-*^ and *Th-Cre*^*+/-*^*;SNCA*^*+/-*^ mice (Fig 1A). This approach allows the stimulation of LC-NE neuronal subpopulations at the onset of behavioral abnormalities, and the characterization of this manipulation of the propagation of synucleinopathy and TH neuronal cell loss in SNc. Successful targeting of LC-NE neurons was evaluated by immunohistochemistry (Fig 1B) and animals without mCherry signal on both sides were excluded. This virus was predominantly localized to presynaptic TH-positive neurons localized in the LC (Fig 1C). Additionally, mCherry axons originating from the LC were observed in the SNc and ventral tegmental area (VTA) regions (Fig 1D) confirming previous observations that LC and SNc are in tight communication [22, 37, 38]. We tested the response of the excitatory hM3Dq receptor by stimulating the animals with clozapine-N-oxide (CNO), an inert compound targeting the M3D receptor and leading to activation of phospholipase C cascade and burst-like firing of neurons [39, 40]. CNO injection led to strong cFOS immunoreactivity specifically in mCherry-expressing LC neurons (Fig 1E).

At 9 months of age, we performed chronic activation of these neurons using osmotic pumps diffusing CNO for six weeks. To rule out whether CNO had any effect on animal behavior, metabolism or brain histopathology, we examined CNO’s potential effects by including saline-treated animals. We characterized the following groups: WT^Control^ (ssaline-treated Th-Cre^+^;SNCA^-^); WT^LC^ (CNO-treated Th-Cre^+^;SNCA^-^); SNCA^Control^ (CNO-treated Th-Cre^-^;SNCA^+^) and SNCA^LC^ (CNO-treated Th-Cre^+^;SNCA^+^).

**Fig 2:**
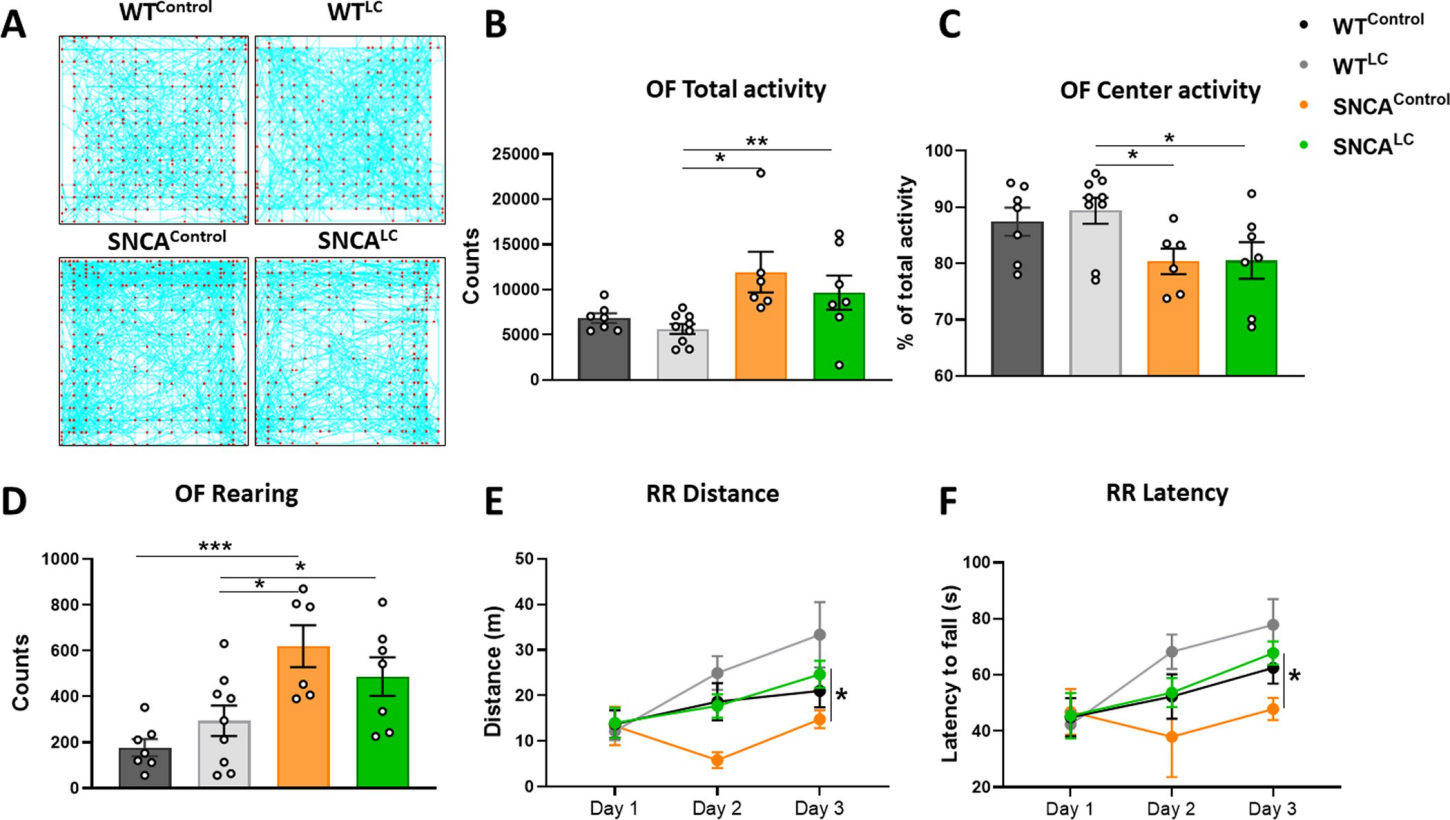
Improved motor function after 6 weeks of LC-NE stimulation. A- Representative track (blue) and rearing (red dots) patterns of all groups in open field test. B- Total activity, percentage of center activity, and rearing in open field test (n = 6–9). C- Distance and latency to fall over the period of 3 days in rotarod test (n = 6–9). ***p<0.0001, **p<0.001, *p<0.05, all values denote means ± SEM.

To determine the contribution of LC-NE neurons to motor dysfunction, mouse motor behavior was assessed six weeks after initiating chronic LC-NE stimulation. As expected, open field (OF) testing revealed that SNCA^Control^ mice presented early-stage disease phenotypes, including higher spontaneous activity (F _(3, 25)_ = 4.204, p = .0154), reduced exploration of the central space of the OF arena (F _(3, 25)_ = 3.220, p = 0.398) and increasing rearing (F _(3, 25)_ = 7.017, p = .0014) compared to WT groups (Fig 2A–2C). CNO treatment had no effect on WT mice. Compared with WT groups, SNCA^LC^ mice also displayed increased total activity and avoided the center of the arena (Fig 2C) but had a greater degree of variability in their exploratory behavior compared to SNCA^Control^ mice. After OF, animals were challenged to a three-day training paradigm on the rotarod (RR). RR testing revealed reduced endurance and motor coordination in SNCA^Control^ compared to WT animals in forced motor activity (latency: F _(1.880, 42.29)_ = 15.48, p < .0001 for factor time, distance: F _(1.811, 54.33)_ = 16.42, p < .0001 for factor time)(Fig 2E and 2F). SNCA^Control^ did not improve their distance or latency performance over the course of the three days compared to other groups. Remarkably, SNCA^LC^ animals showed improved RR distance and latency performance compared to SNCA^Control^, with a phenotype similar to WT animals (Fig 2E and 2F). Taken together, these results suggest that high LC-NE neuronal activity ameliorates motor coordination associated with synucleinopathy.

### Reduced dopaminergic neurodegeneration in the substancia nigra of LC-NE stimulated animals

**Fig 3:**
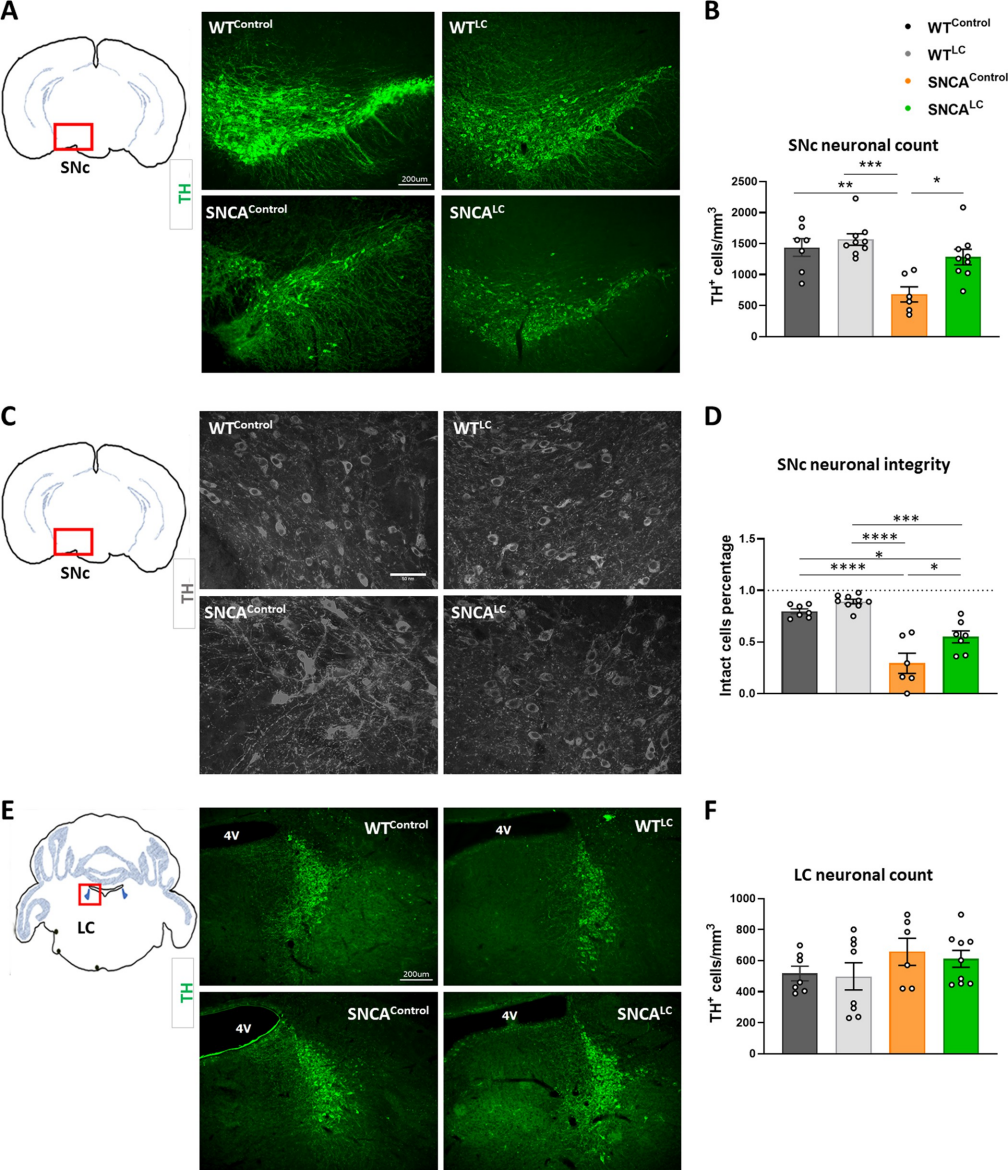
Neuroprotective effect of LC-NE action on SNc dopaminergic neurons. A-Representative images of TH staining in SNc from all groups, TH (green), scale 200μm. B-TH-positive cells count in SNc (n = 6–9). C- Representative absolute-eigen images of TH staining from all groups in SNc, scale 50μm. D- Quantification of TH^+^ cellular morphology integrity in SNc (n = 6–9). E- Representative images of TH staining in LC from all groups, TH (green), scale 200μm.G- Quantification of TH-positive cells in LC (n = 6–9). ****p<0.00001, ***p<0.0001, **p<0.001, *p<0.05, all values denote means ± SEM.

An important feature of α-syn transgenic mice from the line G2-3(A53T) is the concomitant appearance of motor defects and neurological symptoms [29]. We therefore examined the cytopathology associated with the neurological abnormalities in the brains of all mice shortly after behavioral analysis by using a variety of approaches. Brain immunohistochemistry for TH-positive (TH^+^) neurons revealed that CNO treatment in the absence of synucleinopathy (WT^LC^) had no impact on DA neuronal density in the SNc compared with saline treatment (WT^Control^, Fig 3A and 3B). However, in the presence of human α-syn A53T (SNCA^Control^), CNO treatment led to extensive DA neuron depletion in the SNc of mice harboring the α-syn transgene compared to WT groups (F_(3, 27)_ = 8.996, p = .0003) (Fig 3A and 3B). Remarkably, it is established that this model shows little neurodegeneration over time [29]. We confirmed that α-syn A53T overexpressing mice treated with saline did not present neurodegenerative pathology or phenotypes at this stage (S1 Fig). Remarkably, six weeks of hM3D stimulation protected SNCA^LC^ animals from DA neuron depletion despite harboring α-syn A53T overexpression (Fig 3A and 3B). Higher magnification analysis revealed that the neuritic outgrowths of TH neurons from SNCA^Control^ mice were characterized by aberrant DA cellular morphology, abnormal sprouting and reduced processes (Fig 3C). However, DA neurons from SNCA^LC^ mice were morphologically more similar to WT, with improved cytoplasmic and nuclear morphology (Fig 3C). As a result, the percentage of intact cells was higher in the SNCA^LC^ group compared to SNCA^Control^ (F _(3, 25)_ = 25.83, p < .0001) (Fig 3D).

An important observation in post-mortem human PD brain pathology is the concomitant degeneration of LC-NE neurons together with SN-DA neurons. However, the exact timing of this degenerative process remains vague. Early loss of LC neurons in synucleinopathy model would antagonize the ability of the hM3D activation to promote sufficient stimulation of LC-NE neurons. We therefore examined whether TH-positive neuronal count was altered in our model. We found that LC-NE neurons remained preserved in SNCA^Control^ and SNCA^LC^ compared to WT groups (Fig 3E and 3F). Importantly, chronic stimulation of LC neurons did not cause apoptosis of these neurons in either WT or SNCA genetic background. This shows that at 11 months of age, SNCA animals experience DA neuronal loss but retain their LC-NE neurons. However, more discrete changes in LC-NE neuron physiology such as alteration in neurotransmitter release may occur earlier in the disease setting.

### LC activation does not mitigate α-syn aggregation or neuroinflammation in the brain

**Fig 4:**
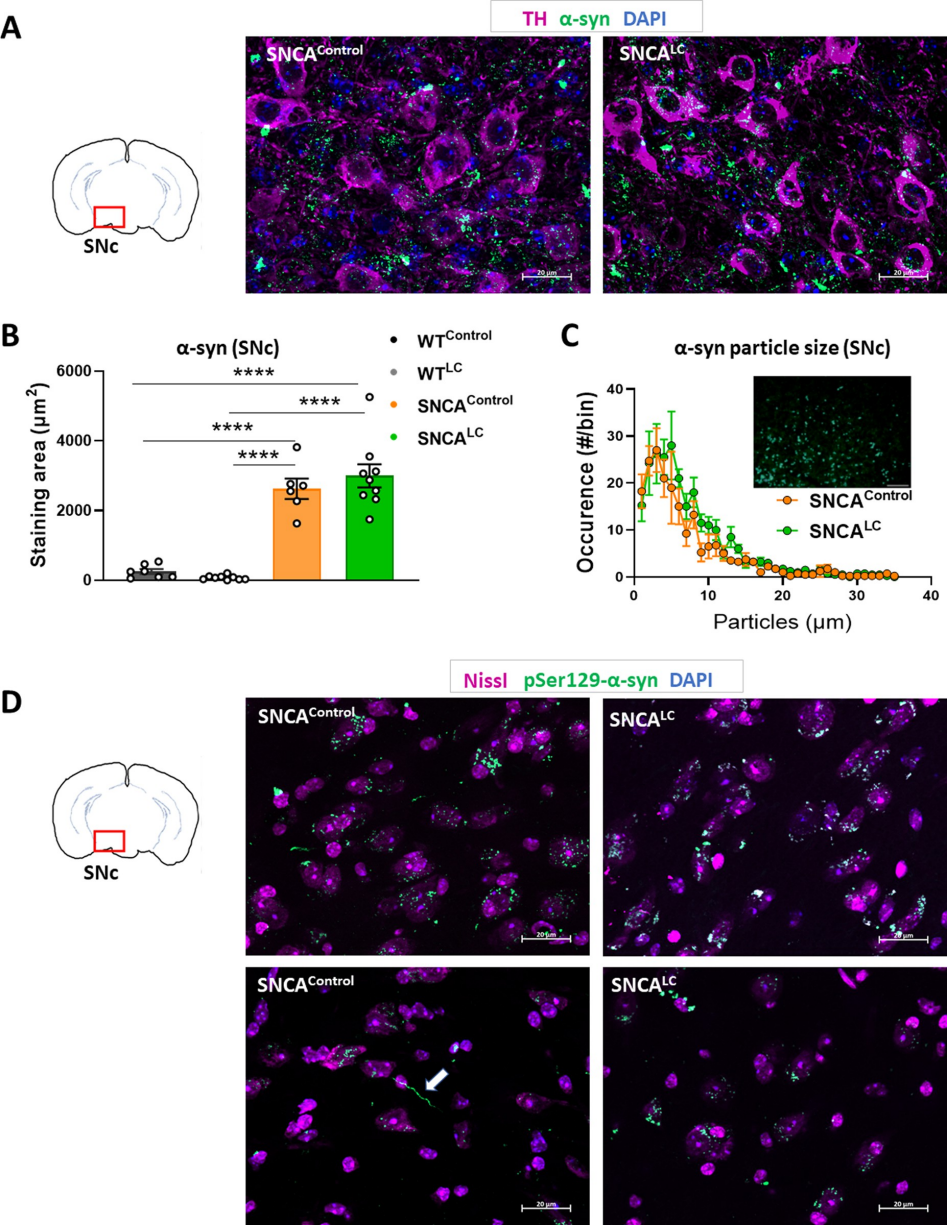
LC-NE activation does not mitigate α-synuclein aggregation. A- Immunohistochemistry against α-syn and TH in SNc of SNCA^Control^ and SNCA^LC^ mice, TH (purple), α-syn (green), DAPI (blue), scale bar 20μm. B- Quantification of the total area of α-syn positive staining in the SNc (n = 6–9). C- Analysis of α-syn particle size distribution in SNCA^Control^ and SNCA^LC^ mice (n = 4–6), right upper panel: representative image used in the analysis, obtained by BX-Z Analyzer software, scale 50μm. D- Immunohistochemistry against pSer129-αSyn from all groups, pSer129-αSyn (green), Nissl (magenta), DAPI (blue), scale bar 20μm. Arrow denotes Lewy neurites. ****p<0.00001, all values denote means ± SEM.

We investigated whether pathological accumulation of α-syn was reduced in neuronal cell bodies and neurites in the midbrain of SNCA^LC^ mice. Abnormal accumulation of α-syn in perikarya and neurites of SNc neurons was visible in both SNCA^Control^ and SNCA^LC^ mice (Fig 4A). Detailed quantification revealed a similar level of α-syn accumulation with higher variability in SNCA^LC^ animals (F_(3, 28)_ = 53.02, p < .0001) (Fig 4B). Considering that conformational changes of α-syn from monomers to oligomers and fibrils are signs of onset and progression of PD [41], we investigated the size of α-syn particles amongst the SNCA^+^ groups (Fig 4C). Not only was the range of α-syn particle size similar between SNCA^Control^ and SNCA^LC^ groups, but increased LC-NE activity did not alleviate the formation of larger particles which could underlie toxic oligomeric forms of α-syn.

To evaluate the presence of LB in the SNc, we digested the sections with proteinase K and stained for pSer129 α-syn. Both SNCA^Control^ and SNCA^LC^ groups displayed densely labelled pSer129 α-syn positive cells in the SNc which resemble LB observed in human PD brains [42]. We did not observe differences in the amount of pSer129 α-syn LB between SNCA^Control^ and SNCA^LC^ groups (Fig 4D). However, we were able to visualize sparse dystrophic neurites in SNCA^Control^ but failed to see them in SNCA^LC^ animals.

We observed correlations between SNc α-syn staining area and RR performance in SNCA^LC^ animals (r(9) = -.8586, p = .0030 for latency to fall, r(9) = -.7744, p = .0143 for the distance) (S2 Fig). Marked negative correlations between these factors suggest that RR performance faithfully informs about α-syn aggregation status. The high variance of the SNCA^LC^ group may denote the degree of experimental surgery success in our cohort, as well as heterogeneity of disease progression before treatment or treatment responsiveness. As in SNc, we also analyzed human α-syn immunofluorescence in the LC and fail to note any difference between SNCA^Control^ and SNCA^LC^ in that region (F _(3, 28)_ = 22.11, p < .0001) S2 Fig).

Monomeric or aggregated α-syn is phagocytosed by microglia, brain-resident macrophages, and leads to their activation [1]. Brain NE is likely to influence microglial recruitment as adrenergic receptors are present on these cells [43] and LC lesion assays have been found to cause DA degeneration and microglial recruitment in the midbrain [28, 44]. We therefore investigated whether microglia were activated upon LC-NE stimulation. We analyzed the number and size of Iba1^+^ microglia in the SNc. Microglial area was similar among SNCA groups (S3 Fig). Compared with WT animals, SNCA animals’ microglia appeared activated, with a larger soma (F _(3, 26)_ = 6.024, p = .0029) and reduced endpoints (F _(3, 25)_ = 2.695, p = .0676) and processes F _(3, 23)_ = 4.247, p = .0158). Microglia from SNCA^LC^ animals did not exhibit lower activation than SNCA^Control^. In the LC region, iba1 immunoreactivity was also similar amongst all animals (S3 Fig).These data suggest that activating LC-NE neurons in α-syn overexpressing mice does not reduce neuroinflammation associated with synocleinopathy in SNc. Our results suggest that the neuroprotective effect of activating LC-NE neurons in SNCA mice may be uncoupled from pathological α-syn accumulation and microgliosis.

### LC-NE activation promotes insulin resistance in human A53T mutant α-syn expressing mice

**Fig 5:**
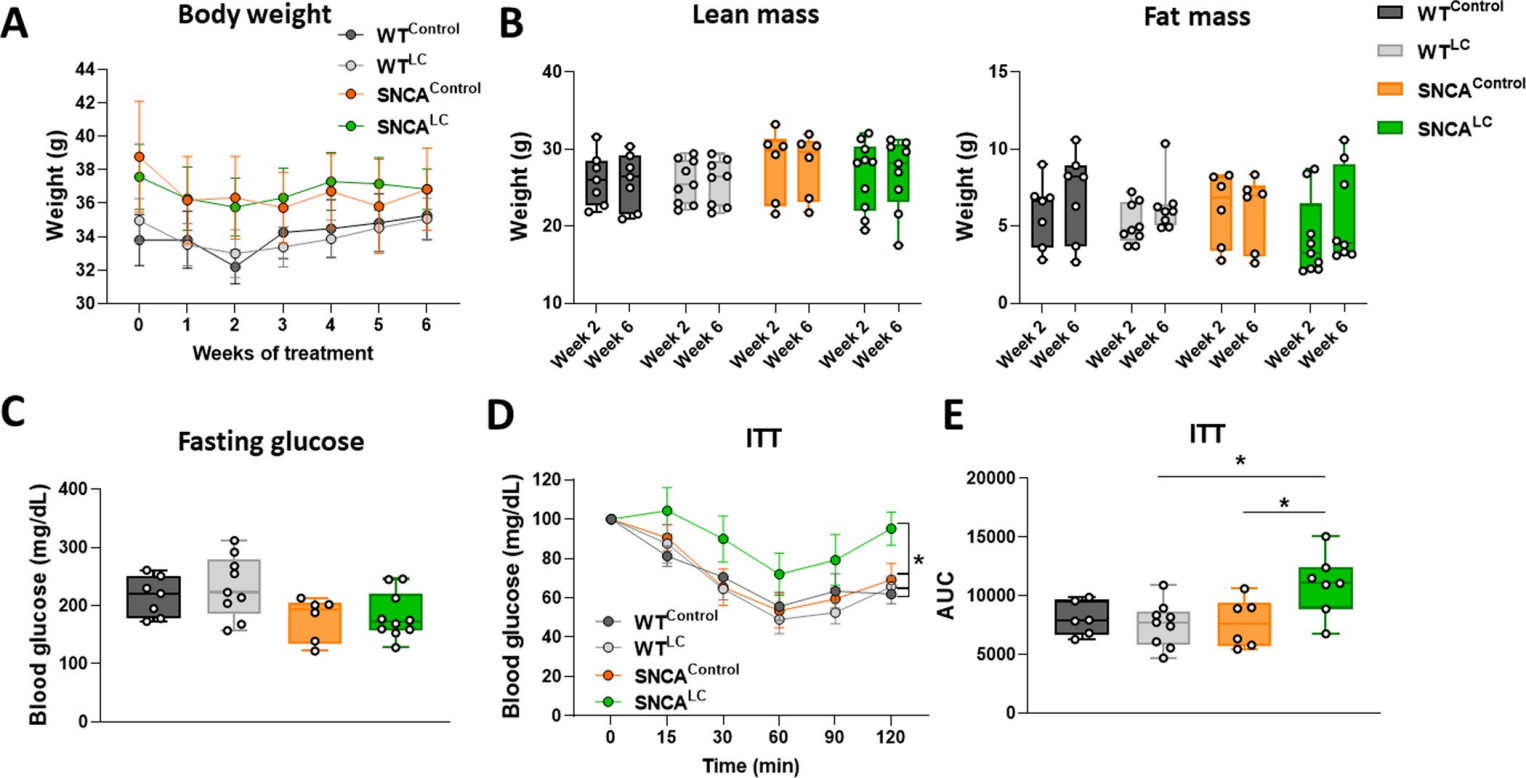
LC-NE chronic stimulation promotes systemic insulin resistance in α-syn overexpressing animals. A- Changes in body weight over 6 weeks during CNO treatment, (n = 6–9). B- Lean and fat mass at weeks 2 and 6 of chronic LC-NE stimulation (n = 6–9). C-Fasting blood glucose levels on week 6 of LC-NE chronic stimulation (n = 6–9). D- Insulin tolerance test performed at week 6 of CNO treatment, (n = 6–9). E- Insulin tolerance testing area under the curve analysis 6 weeks after beginning of the CNO treatment (n = 6–9). *p<0.05, all values denote means ± SEM.

An established consequence of NE infusion is to cause insulin resistance (IR) [45]. Insulin has pleiotropic effects, ranging from controlling peripheral blood glucose level, to modulating energy homeostasis (through hypothalamic arcuate neurons), to neuroprotection (via regulation of neuronal survival and growth), to DA transmission, and finally, to synaptic maintenance [46]. IR has been widely observed in PD patients, but conflicting reports exist regarding the connection between IR and outcome of disease progression, such as dementia [47–49]. Because of these links between NE and IR, we aimed at investigating whether chronic stimulation of LC-NE neurons was associated with IR and impaired glucose homeostasis. We monitored animals’ body weight throughout the study. As noted in WT animals, chronic CNO treatment did not cause weight loss compared to saline controls (Fig 5A). Mice overexpressing α-syn showed a trend towards increased weight compared with WT groups, with SNCA^LC^ animals being indistinguishable from SNCA^Control^ animals. No changes in lean mass and fat mass were found in the groups (Fig 5B). Fasting blood glucose was unchanged in SNCA compared to WT groups (Fig 5C). We found that SNCA^LC^ mice were insulin resistant after 6 weeks of LC-NE stimulation when challenged with an insulin sensitivity test (F _(3, 24)_ = 4.349. p = .0139) (Fig 5D and 5E). However, this IR was compensated as neither weight or fasting glucose were altered in SNCA^LC^ mice.

Because of the presence of systemic IR in SNCA^LC^ mice, we used indirect calorimetry to assess if energy homeostasis was altered in our experimental cohorts. Within two weeks of chronic CNO treatment, SNCA^LC^ mice appeared slightly hypometabolic, as measured by oxygen consumption and heat production (F _(3, 26)_ = 2.068, p = 0.1301) (S4 Fig). This reduced energy expenditure persisted over time (F _(3, 26)_ = 2.536, p = .0787) and was not due to decreased feeding, water intake or physical activity. Taken together, enhanced noradrenergic action in the α-syn A53T overexpression background promotes peripheral IR, without overt side effects on weight gain or blood glucose levels.

### NE underlies the neuroprotective action of the LC on SN-DA neurons

Previously, NE has been shown to exert a trophic effect on DA neurons in embryonic rat mesencephalic cultures [50]. We next investigated whether NE was responsible for improved DA neuron survival upon activation of LC-NE neurons. We therefore examined the effects of NE on survival of DA neurons in SN-VTA midbrains cultures isolated from mouse neonates. In a neuronal medium consisting of DMEM-F12 supplemented with Glutamax, B27 with antioxidants, NE had no beneficial effects on neuron survival or neurite sprouting (S5 Fig). Surprisingly, addition of the standard antioxidant Trolox to the medium containing antioxidants was slightly toxic to the culture (F _(3, 4)_ = 6.833, p = .0472). However, when antioxidants were omitted from the medium (S5 Fig), NE exerted a dose-dependent increase in DA neuron survival (1–10μM), as measured after 4 days in culture. NE also led to trophic effects on total neurite length (F _(3, 231)_ = 9.101, p < .0001), mean neurite length (F _(3, 231)_ = 12.59, p < .0001), and dendritic roots (i.e., neurite emergence points on the cell body) outgrowth (F _(3, 231)_ = 3.671, p < .0001). The lowest concentration tested (1 μM) was sufficient to promote increased DA neuron survival, mean and total neurite length. We therefore used this concentration in the subsequent experiments. Supplying NE to the culture media increased survival after 4 days (F _(2, 3)_ = 11.02, p = 0.0415), but not after 8 days, where neuronal depletion was almost complete (S6 Fig). Surviving DA neurons at day 4 showed a dramatic gain in sprouting with NE, which was superior to the neurotrophic effects of 10μM GDNF (F _(2, 209)_ = 18.42, p < .0001) (mean neurite length, S6 Fig).

**Fig 6:**
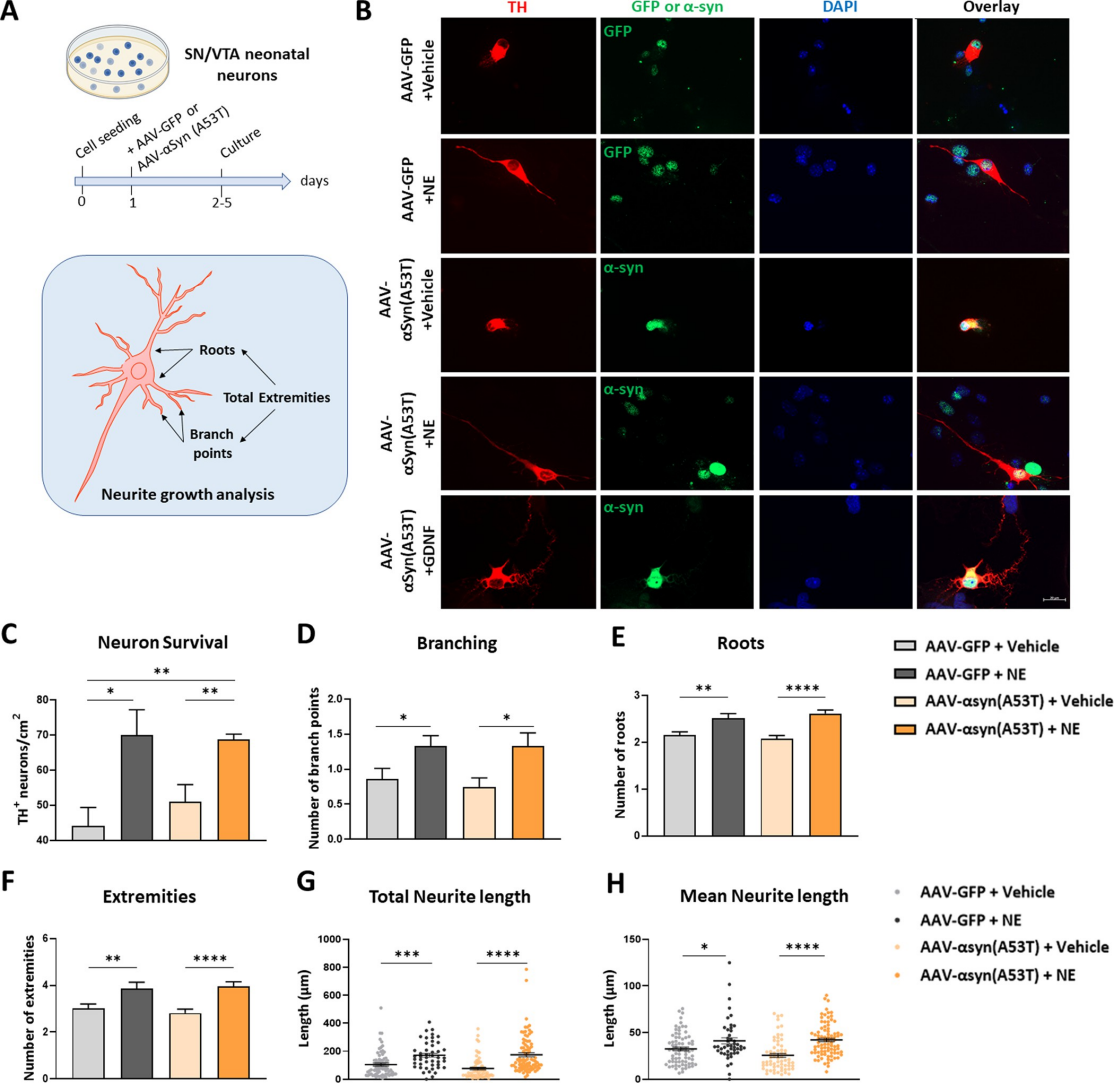
Neuroprotective role of NE in AAV-α-synuclein transduced primary DA cell culture. A- Time line for primary mouse ventral midbrain dopaminergic neurons tissue culture and representation of neurite analysis showing examples of roots, branch points, and total extremities. B- Representative images of dopaminergic neurons infected with AAV-hSynA53T α-synuclein or AAV-GFP, supplemented with vehicle, NE, or GDNF and immunostained with TH (red), GFP (green) or α-synuclein (green) with DAPI nuclear stain (blue), scale bar 20 μm. C- TH neurons survival. D-H- Neurite growth analysis quantifying branching, root number, extremities and neurite length. ****p<0.00001, ***p<0.0001, **p<0.001, *p<0.05, all values denote means ± SEM.

Having established that NE exerts neuroprotective effects on cultured DA neurons, we then examined whether these beneficial effects were maintained in the presence of pathogenic α-syn. We infected DA neuron cultures with AAV1/2-hu-A53T-α-syn or control virus AAV1/2-GFP and maintained these cells for 4 days. We measured the survival, and the neurotrophic effects of NE on DA neurons’ neurite length, number of roots, branch points and total number of extremities (Fig 6A). Immunofluorescent labelling of GFP and α-syn were observed in TH-positive DA neurons (Fig 6B). Of note, A53T-α-syn expression did not lead to reduced neuronal survival compared to GFP after 5 days in culture without antioxidants, or after 8 days in medium containing antioxidants (not shown). NE (1μM) showed beneficial effect on survival and parameters of neuronal growth as treatment increased survival (Fig 6C) and numbers of branch points (F _(3, 295)_ = 4.621 p = 0.0036), roots (F _(3, 295)_ = 11.33, p < .0001), extremities (F _(3, 295)_ = 8.931, p < .0001) and neuronal length (F _(3, 278)_ = 12.04, p < .0001) (Fig 6D–6H) in all DA neurons. The beneficial effect of NE was observed independently in both control (GFP) and A53T-α-syn expressing DA neurons. NE treatment provoked a gain in survival in neurons expressing A53T-α-syn, compared to vehicle or GDNF treatment (S7 Fig). Interestingly, GDNF did not significantly improve survival of DA neurons infected with AAV1/2-hu-A53T-α-syn,despite trophic effects on neurite sprouting and increased survival in wild-type neurons. Therefore, NE possesses unique neuroprotective properties to promote survival of DA neurons in the presence of pathogenic α-syn.

## Discussion

Several lines of evidence suggest that NE may be protective in PD. Negative correlations between noradrenergic neuronal projections and DA depletion in the Nucleus Accumbens have been observed in post-mortem PD human brains [51], indicating that alterations in the release of central NE may directly influence the rate of PD progression. In mouse models, increasing synaptic NE by genetic deletion or pharmacological blockade of the NE transporter (NET) confers resistance to chemical depletion of DA neurons using MPTP toxicity [17, 18]. In line with these observations, mice mutant for DBH unable to synthesize NE developed motor deficits [20]. As a mouse model to investigate the effects of NE on pathogenic α-syn was missing, we investigated the impact of increasing LC-NE activity in mice overexpressing human A53T α-syn. We applied the DREADD technology to temporally activate these neurons at an early stage of pathogenesis with the goal to generate an adult-onset model of enhanced LC-NE activity. Previous attempts to enhance NE action have targeted neonatal genetic deletions of NET have provided strong evidence for a role of NE in DA neuron survival [17, 18]. Our results demonstrate that chronic stimulation of LC-NE neurons at the onset of SNc neurodegeneration protects against PD pathogenesis by improving motor coordination and reducing DA neuronal death (Figs 2 and 3). However, we find that this improved DA neuron survival is not due to reduced α-syn aggregation in the SNc region (Fig 4). The mechanisms responsible for preserving DA neurons’ integrity upon higher NE release remain to be identified. Lesion models of LC-NE neurons have found an increased microglial response in the CNS associated with SNc neurodegeneration [28, 44]. Interestingly, we do not find reduced microgliosis upon higher LC-NE activity, arguing that the beneficial effects observed may be due to direct action of LC-NE on SN-DA neuron survival. The cellular effects of NE on DA neurons remain poorly understood and may be critical to a better understanding of PD pathophysiology.

An important question raised by post-mortem PD brain analysis is whether LC-NE neurons degenerate earlier than SNc-DA neurons in PD and cause their demise [14, 15, 52]. Here we do not find concomitant neurodegeneration of LC-NE neurons with SNc-DA neurons in α-syn overexpressing mice (Fig 3E and 3F), arguing that LC-NE neurons remain preserved in our model of synucleinopathy. Therefore, our results suggest that LC lesion assays do not recapitulate α-syn disease progression and are in line with human clinical data, showing little damage to the LC in early PD [52]. We also do not find reactive microglia accumulation in the LC associated with synucleinopathy (S3 Fig).

In accordance with our findings, chronic CNO delivery is not associated with SNc neurodegenerative effects in the absence of synucleinopathy [53] (S1 Fig). However, we find that chronic CNO treatment specifically promotes neurodegeneration of TH SNc neurons in α-syn A53(T) mice. The mechanisms responsible for this neurodegeneration remain to be identified.

Because NE has been linked with IR in humans [45, 54], we asked if high NE may connect metabolic dysfunction, IR, and α-syn pathology in PD. Our results clearly demonstrate that high LC-NE activity drives IR in α-syn overexpressing mice but not in a wild-type background (Fig 6D and 6E). Therefore, our data suggest that the neuroprotective effect of NE on DA neurons can be uncoupled from its action on IR. Because of the short-term nature of our DREADD model, we could not investigate if life-long IR in mice could reverse the protective effects of NE on SN-DA neuron survival. One important finding in this model is the trend towards increased microgliosis observed in the midbrain of SNCA^LC^ mice, which may be a consequence of brain IR and could become pathogenic to synapses and induce neurotoxicity over time [55]. Metabolic syndrome has been proposed as a potential catalyzer for neurodegenerative diseases such as Alzheimer’s disease and PD [56, 57]. The anti-diabetic drug exanitide, which activates GLP-1 receptors was shown to have a beneficial impact on motor symptoms in a cohort of 60 moderate stage PD patients [58]. Remarkably, a high prevalence of IR and/or insulin deficiency has been monitored in PD patients [49] but does not necessarily correlate with their PD motor, non-motor and cognitive scores. Whether IR can accelerate PD is a fundamental question, but remains of debate in clinical studies [49, 59, 60]. In mice, brain IR altered DA turnover and caused anxiety and depression [61], without inducing neurodegeneration of DA neurons. To better understand the consequence of prolonged IR on PD pathophysiology, further studies will be required.

Our results strongly support a neuroprotective role for NE on DA neuron fate (Fig 6). The etiology of PD remains complex and is recognized as being the result of multiple insults impairing processes of proteostasis, mitochondrial function and oxidative stress. Much evidence has revealed pathogenic reactive oxygen species (ROS) accumulation causing cellular stress including lipid peroxidation, DNA damage and post-translational protein alterations [62–64]. Weakening of the antioxidative defense system with age has been postulated to underlie PD disease progression. This system is composed of enzymes and factors such as glutathione regulating calcium homeostasis, redox balance and protein folding which play a key protective role in DA neurons. Among these, selonoproteins have been demonstrated to be neuroprotective in mouse models of PD [65–68]. In particular, Selenoprotein T (SELT) protects DA neurons through oxidoreductase activity [66]. Remarkably, we find that absence of antioxidants or NE is detrimental to DA neuron survival in culture (S5 Fig). However, supplementation of the classic antioxidant Trolox together with antioxidant-containing medium reduces survival. The data suggest that the nature and dosage of antioxidants may be of primary importance to DA neuron health.

Taken together, these results demonstrate that the noradrenergic system in the brain influences DA neuron viability through NE release and that manipulation of these neurons is efficient in combatting synucleinopathy-driven DA cell death in the SNc.

## Supporting information

S1 Fig Chronic CNO promotes neurodegenerative phenotypes in the presence of synucleinopathy. A- TH-positive cells count in SNc and LC of mice treated with Saline or CNO (n = 6–9). B- Distance and latency to fall over the period of 3 days in rotarod test (n = 6–9). ***p<0.0001, **p<0.001, *p<0.05, all values denote means ± SEM.
(TIF)

S2 Fig Correlation analysis (SNc) and quantification of α-syn content in LC. A- Distribution plot of Pearson correlation between α-syn aggregation in ventromedial part of SNc and distance crossed (orange) and latency to fall (black) in rotarod task in SNCA^LC^ mice. B- Quantification of total area of α-syn positive staining in the LC (n = 6–10). ****p<0.00001, ***p<0.0001, all values denote means ± SEM.
(TIF)

S3 Fig Neuroinflammation upon α-synuclein aggregation is independent of LC-NE action. A- Representative immunofluorescent images of microglial Iba1 staining in SNc of SNCA^Control^ and SNCA^LC^ mice, Iba1 (magenta), scale bar 50μm. B- Quantification of microglia presence by iba1 staining in SNc (n = 6–8). C, D, E- Analysis of microglia soma size, endpoints per cell and process length per cell within SNc (n = 6–8). F- Representative immunofluorescent images of Iba1 staining in LC of SNCA^Control^ and SNCA^LC^ mice, Iba1 (magenta), scale bar 50μm. G- Quantification of microglia presence by iba1 staining in LC (n = 6–9). **p<0.001, *p<0.05, all values denote means ± SEM.
(TIF)

S4 Fig Chronic stimulation of LC-NE neurons induces metabolic decline in α-syn overexpressing animals. A—Oxygen consumption over the course of 1 day on week of chronic CNO treatment. B,C- Average heat production during light/dark phase obtained by indirect calorimetry on week 2 and week 5 of chronic CNO treatment. D- Cumulative food intake obtained by indirect calorimetry on week 5 of chronic CNO treatment, upper left panel on figure D: food intake on last time point, (n = 6–9). E- Cumulative water intake obtained by indirect calorimetry on week 5 of chronic CNO treatment, upper left panel on figure E: water intake on last time point, (n = 6–9). F- Total activity over the course of 4 days obtained by TSE Phenomater metabolic cage sensors on week 5 of chronic CNO treatment. *p<0.05, all values denote means ± SEM.
(TIF)

S5 Fig Dose-dependent effect of NE on neuronal survival and growth. A- Primary dopaminergic neurons survival in DMEM-F12 supplemented with Glutamax, B27 with antioxidants. B- Primary dopaminergic neurons culture in DMEM-F12 supplemented with Glutamax, B27 without antioxidants and representation of neurite analysis showing examples of roots, branch points, and total extremities. C- NE dose-dependent increase in TH neuron survival and neurite sprouting in medium without antioxidants. ****p<0.00001, ***p<0.0001, *p<0.05, all values denote means ± SEM.
(TIF)

S6 Fig Effect of different types of culture medium on neuronal survival and growth. A—Representative images of TH immunofluorescent staining on day 4 and day 8, respectively, of primary dopaminergic neurons in medium without antioxidants, supplemented with vehicle, NE (1μM) or GDNF (100 ng/mL), TH (green), DAPI (blue), scale bar 50 μm and 100 μm, respectively. B- TH neurons survival at day 4 and day 8, neurite growth analysis at day 4. ****p<0.00001, ***p<0.0001, **p<0.001, *p<0.05, all values denote means ± SEM.
(TIF)

S7 Fig Comparison of neuroprotective effect of NE and GDNF in AAV-α-synuclein transduced primary DA cell culture. A- Relative fold change of dopaminergic neurons survival and B-F neurite growth analysis at day 4. ****p<0.00001, ***p<0.0001, **p<0.001, *p<0.05, all values denote means ± SEM.
(TIF)

S1 File (DOCX)

## Abbreviations

α-syn: α-synuclein
CNS: central nervous system
CNO: Clozapine-N-Oxide
DA: dopaminergic
DBH: dopamine β-hydroxylase
DREADDS: Designer Receptors Exclusively Activated by Designer Drugs
LB: Lewy bodies
LN: Lewy neurites
LC: locus coeruleus complex
hM3D: human muscarinic receptor 3
NE: norepinephrine
PD: Parkinson’s disease
SNc: substantia nigra pars compacta
TH: tyrosine hydroxylase
VTA: ventral tegmental area
