# Supplementary figures and images for: Sustained chemogenetic activation of locus coeruleus norepinephrine neurons promotes dopaminergic neuron survival in synucleinopathy
Source: PLoS One. 2022 Mar 22;17(3):e0263074. doi: 10.1371/journal.pone.0263074 (PMC8939823; doi:10.1371/journal.pone.0263074)

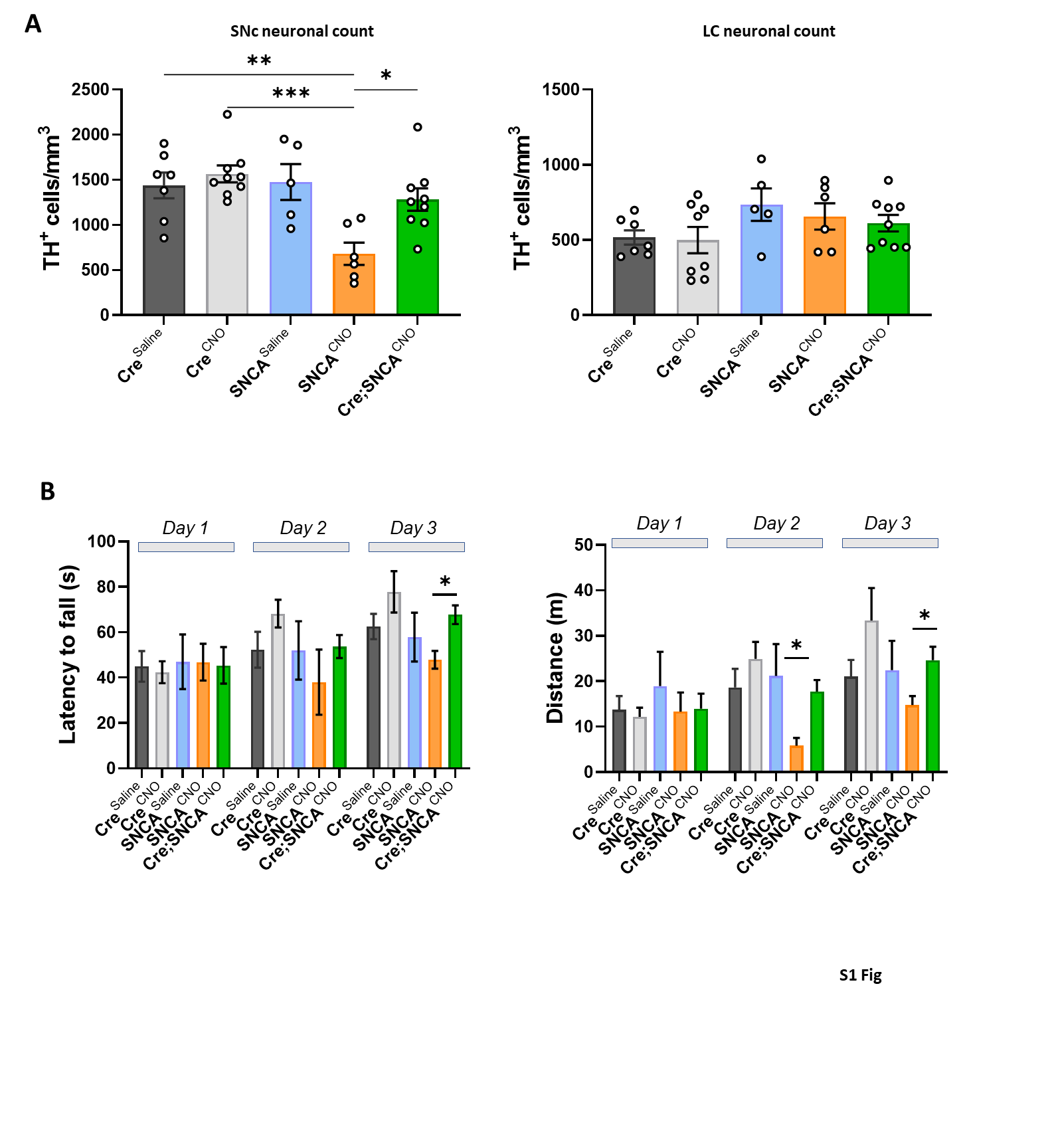

Supplement: S1 Fig — A- TH-positive cells count in SNc and LC of mice treated with Saline or CNO (n = 6–9). B- Distance and latency to fall over the period of 3 days in rotarod test (n = 6–9). ***p<0.0001, **p<0.001, *p<0.05, all values denote means ± SEM. (TIF) [file pone.0263074.s001.TIF]

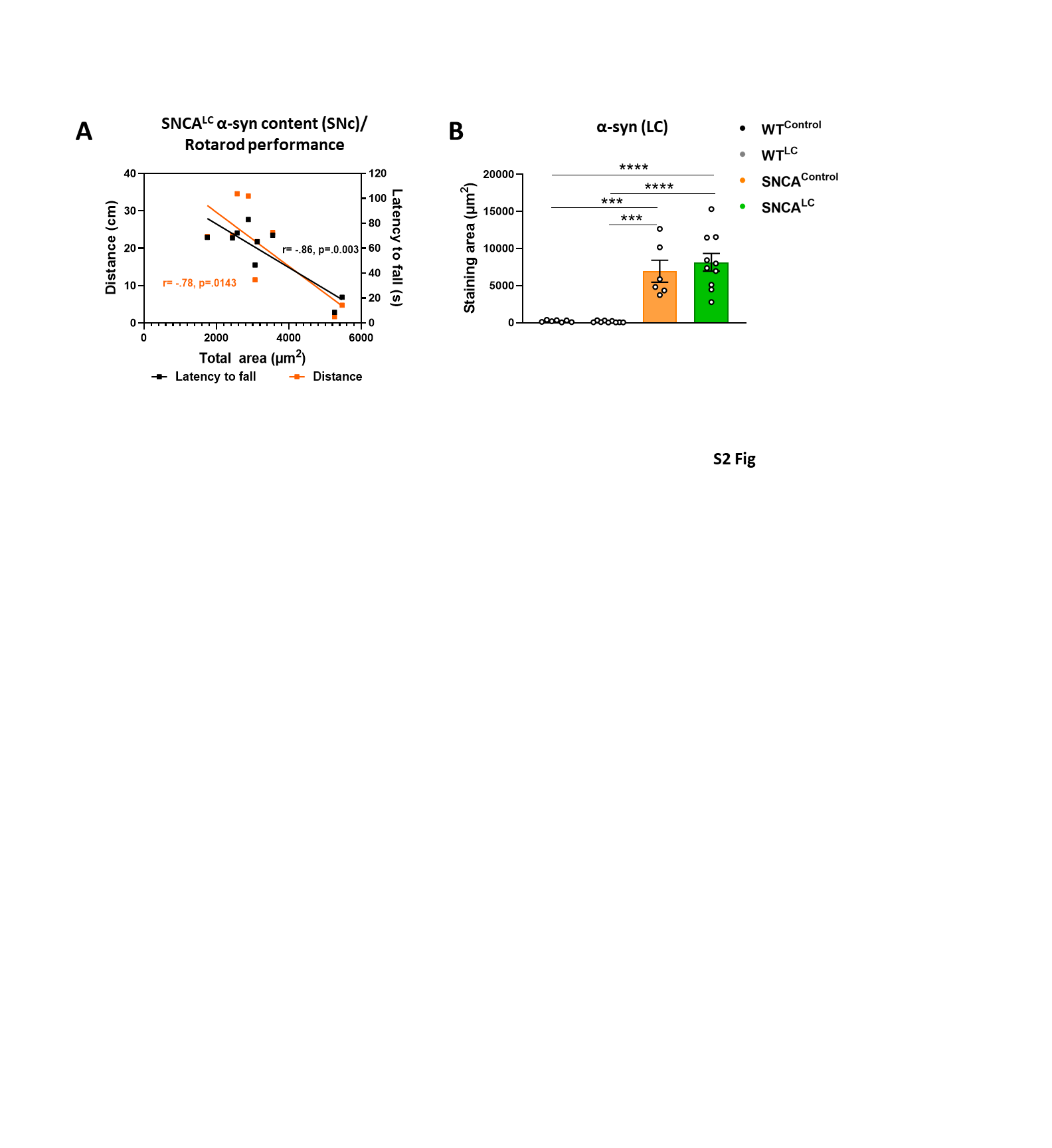

Supplement: S2 Fig — A- Distribution plot of Pearson correlation between α-syn aggregation in ventromedial part of SNc and distance crossed (orange) and latency to fall (black) in rotarod task in SNCALC mice. B- Quantification of total area of α-syn positive staining in the LC (n = 6–10). ****p<0.00001, ***p<0.0001, all values denote means ± SEM. (TIF) [file pone.0263074.s002.TIF]

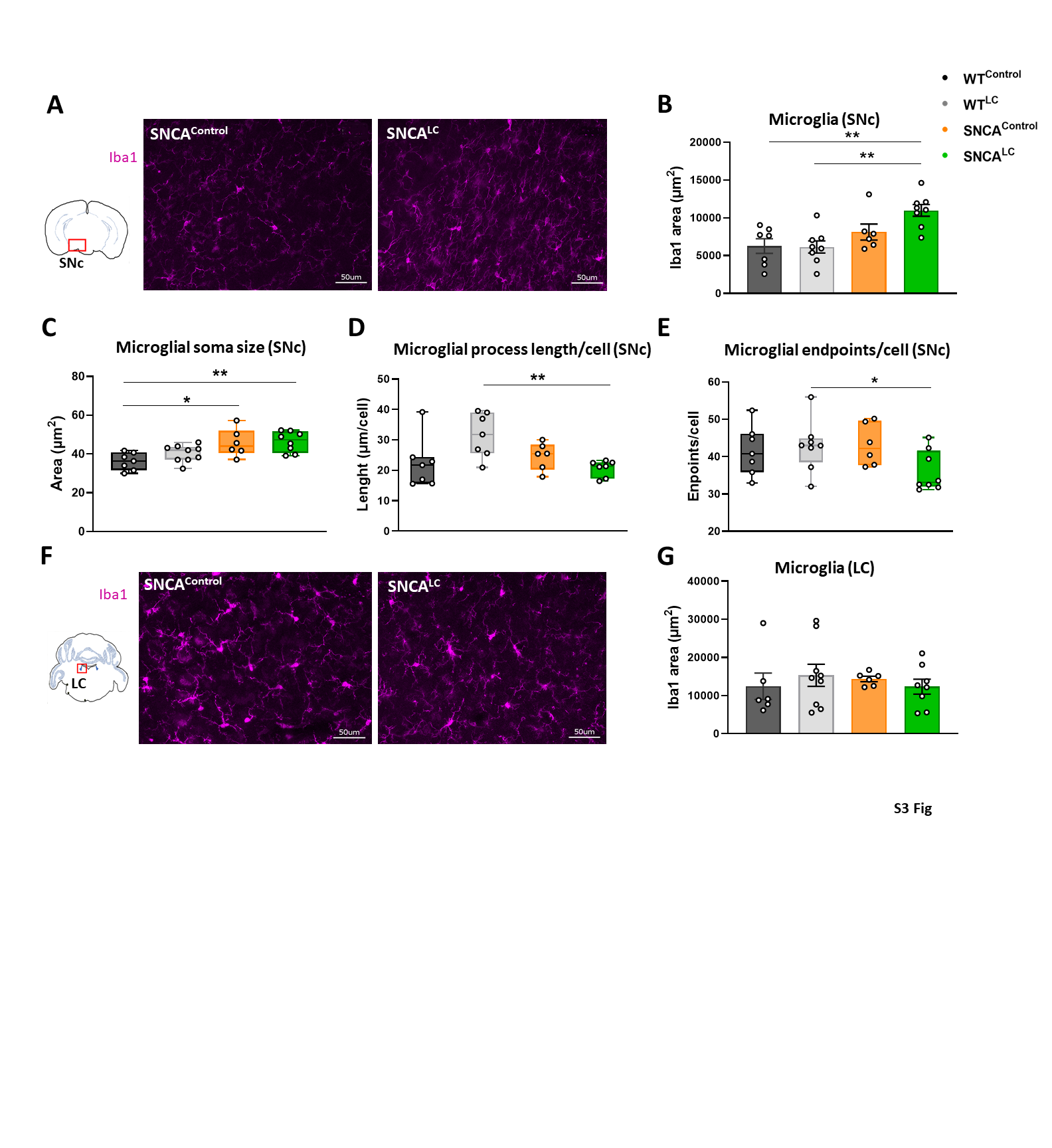

Supplement: S3 Fig — A- Representative immunofluorescent images of microglial Iba1 staining in SNc of SNCAControl and SNCALC mice, Iba1 (magenta), scale bar 50μm. B- Quantification of microglia presence by iba1 staining in SNc (n = 6–8). C, D, E- Analysis of microglia soma size, endpoints per cell and process length per cell within SNc (n = 6–8). F- Representative immunofluorescent images of Iba1 staining in LC of SNCAControl and SNCALC mice, Iba1 (magenta), scale bar 50μm. G- Quantification of microglia presence by iba1 staining in LC (n = 6–9). **p<0.001, *p<0.05, all values denote means ± SEM. (TIF) [file pone.0263074.s003.TIF]

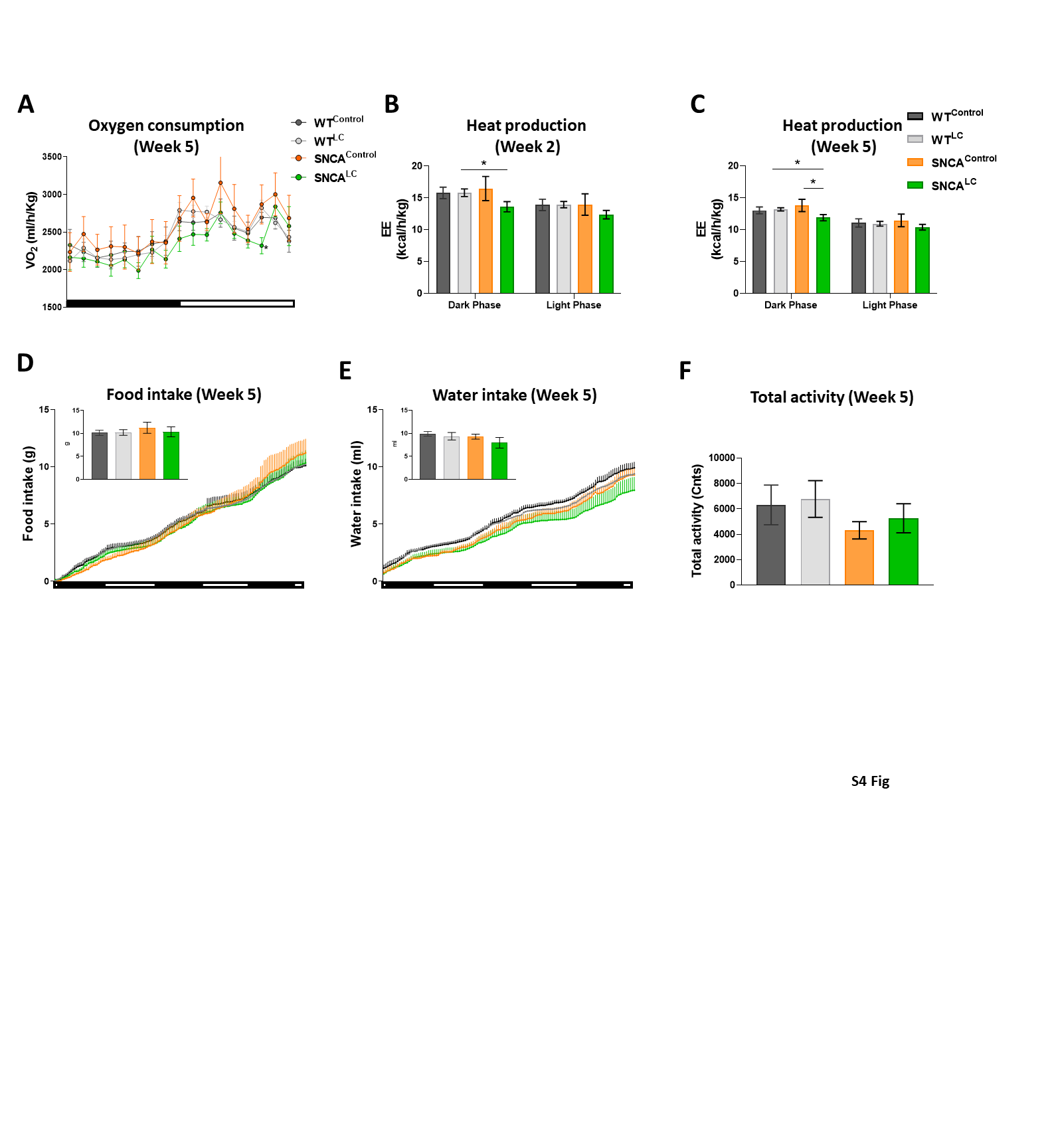

Supplement: S4 Fig — A—Oxygen consumption over the course of 1 day on week of chronic CNO treatment. B,C- Average heat production during light/dark phase obtained by indirect calorimetry on week 2 and week 5 of chronic CNO treatment. D- Cumulative food intake obtained by indirect calorimetry on week 5 of chronic CNO treatment, upper left panel on figure D: food intake on last time point, (n = 6–9). E- Cumulative water intake obtained by indirect calorimetry on week 5 of chronic CNO treatment, upper left panel on figure E: water intake on last time point, (n = 6–9). F- Total activity over the course of 4 days obtained by TSE Phenomater metabolic cage sensors on week 5 of chronic CNO treatment. *p<0.05, all values denote means ± SEM. (TIF) [file pone.0263074.s004.TIF]

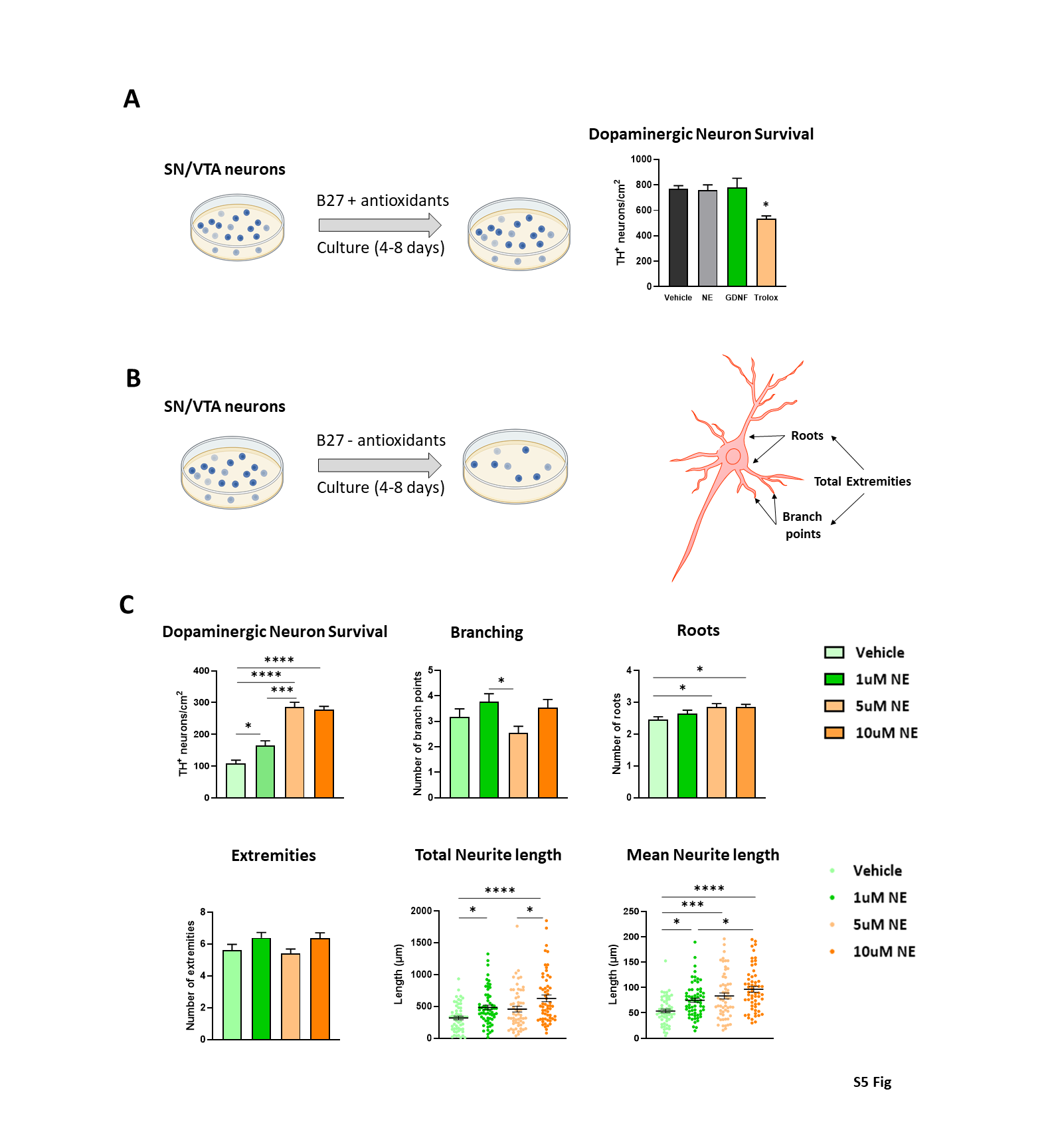

Supplement: S5 Fig — A- Primary dopaminergic neurons survival in DMEM-F12 supplemented with Glutamax, B27 with antioxidants. B- Primary dopaminergic neurons culture in DMEM-F12 supplemented with Glutamax, B27 without antioxidants and representation of neurite analysis showing examples of roots, branch points, and total extremities. C- NE dose-dependent increase in TH neuron survival and neurite sprouting in medium without antioxidants. ****p<0.00001, ***p<0.0001, *p<0.05, all values denote means ± SEM. (TIF) [file pone.0263074.s005.TIF]

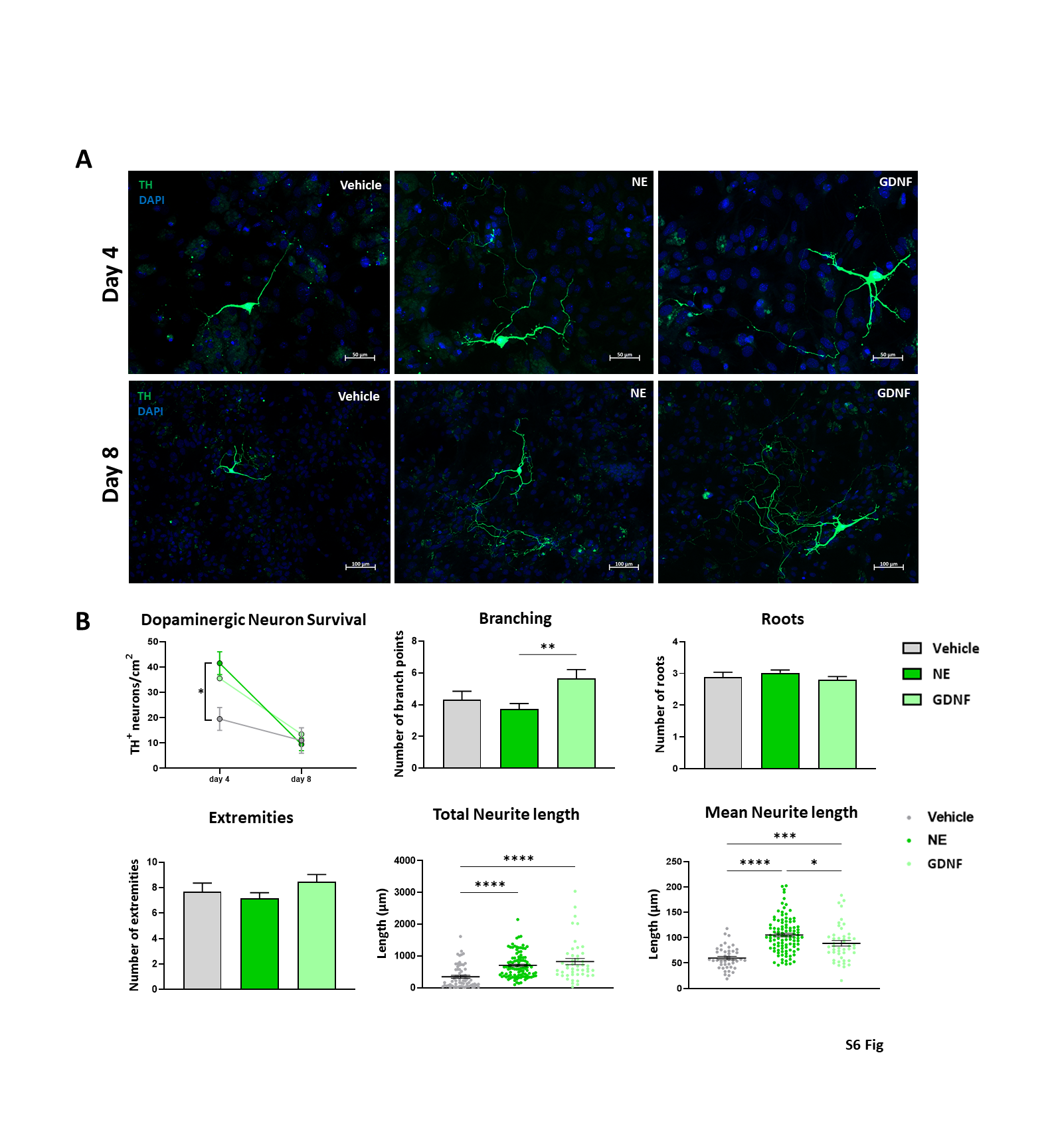

Supplement: S6 Fig — A—Representative images of TH immunofluorescent staining on day 4 and day 8, respectively, of primary dopaminergic neurons in medium without antioxidants, supplemented with vehicle, NE (1μM) or GDNF (100 ng/mL), TH (green), DAPI (blue), scale bar 50 μm and 100 μm, respectively. B- TH neurons survival at day 4 and day 8, neurite growth analysis at day 4. ****p<0.00001, ***p<0.0001, **p<0.001, *p<0.05, all values denote means ± SEM. (TIF) [file pone.0263074.s006.TIF]

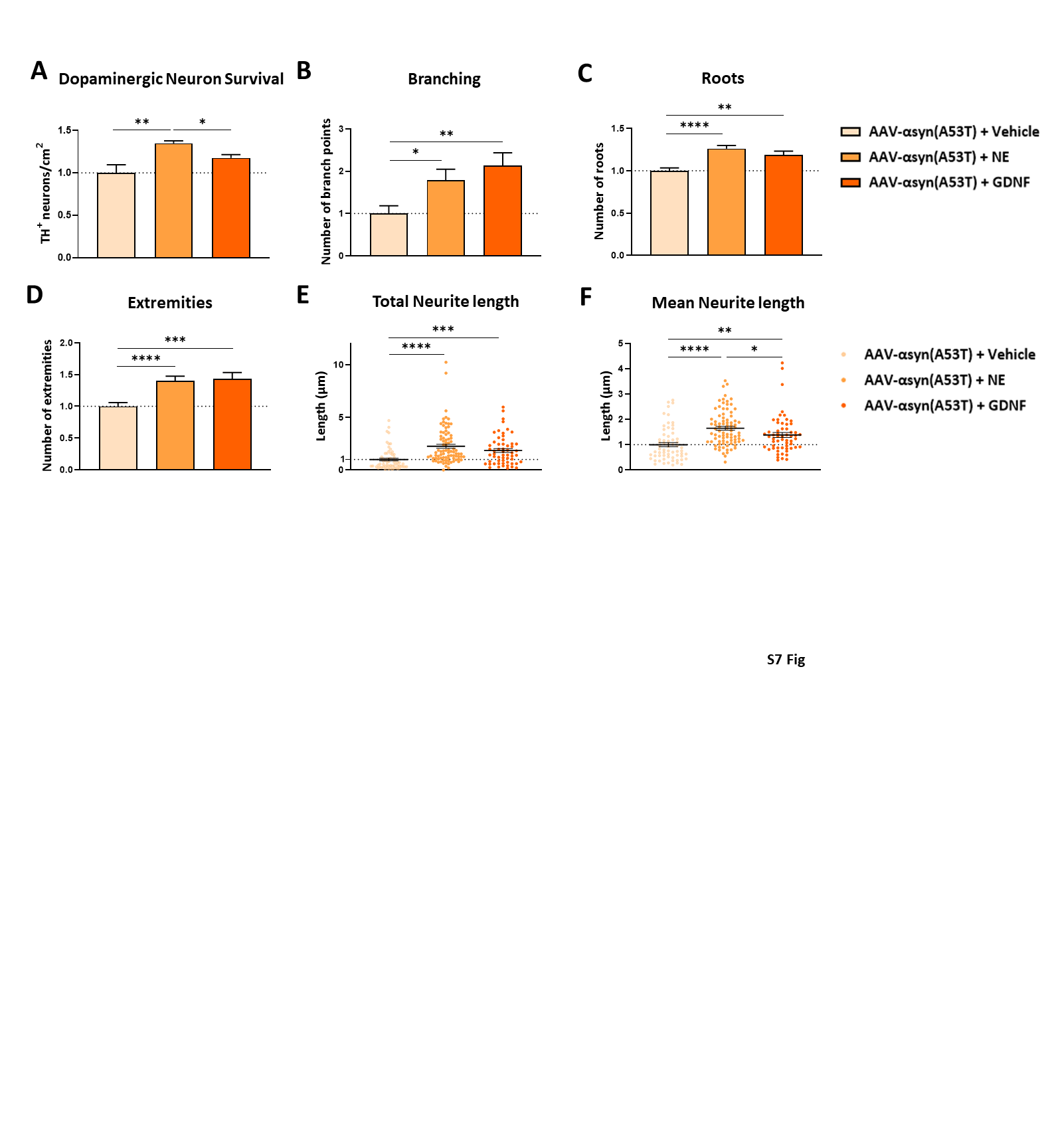

Supplement: S7 Fig — A- Relative fold change of dopaminergic neurons survival and B-F neurite growth analysis at day 4. ****p<0.00001, ***p<0.0001, **p<0.001, *p<0.05, all values denote means ± SEM. (TIF) [file pone.0263074.s007.TIF]
