# Supplementary material for: Sustained chemogenetic activation of locus coeruleus norepinephrine neurons promotes dopaminergic neuron survival in synucleinopathy
Source: PLoS One. 2022 Mar 22;17(3):e0263074. doi: 10.1371/journal.pone.0263074 (PMC8939823; doi:10.1371/journal.pone.0263074)
Supplement: S1 File — (DOCX) [file pone.0263074.s008.docx]

**Supplementary Data.**

**Supplementary figure 1. Chronic CNO promotes neurodegenerative phenotypes in the presence of synucleinopathy.** A- TH-positive cells count in SNc and LC of mice treated with Saline or CNO (n=6-9). B- Distance and latency to fall over the period of 3 days in rotarod test (n=6-9). ***p<0.0001, **p<0.001, *p<0.05, all values denote means ± SEM.


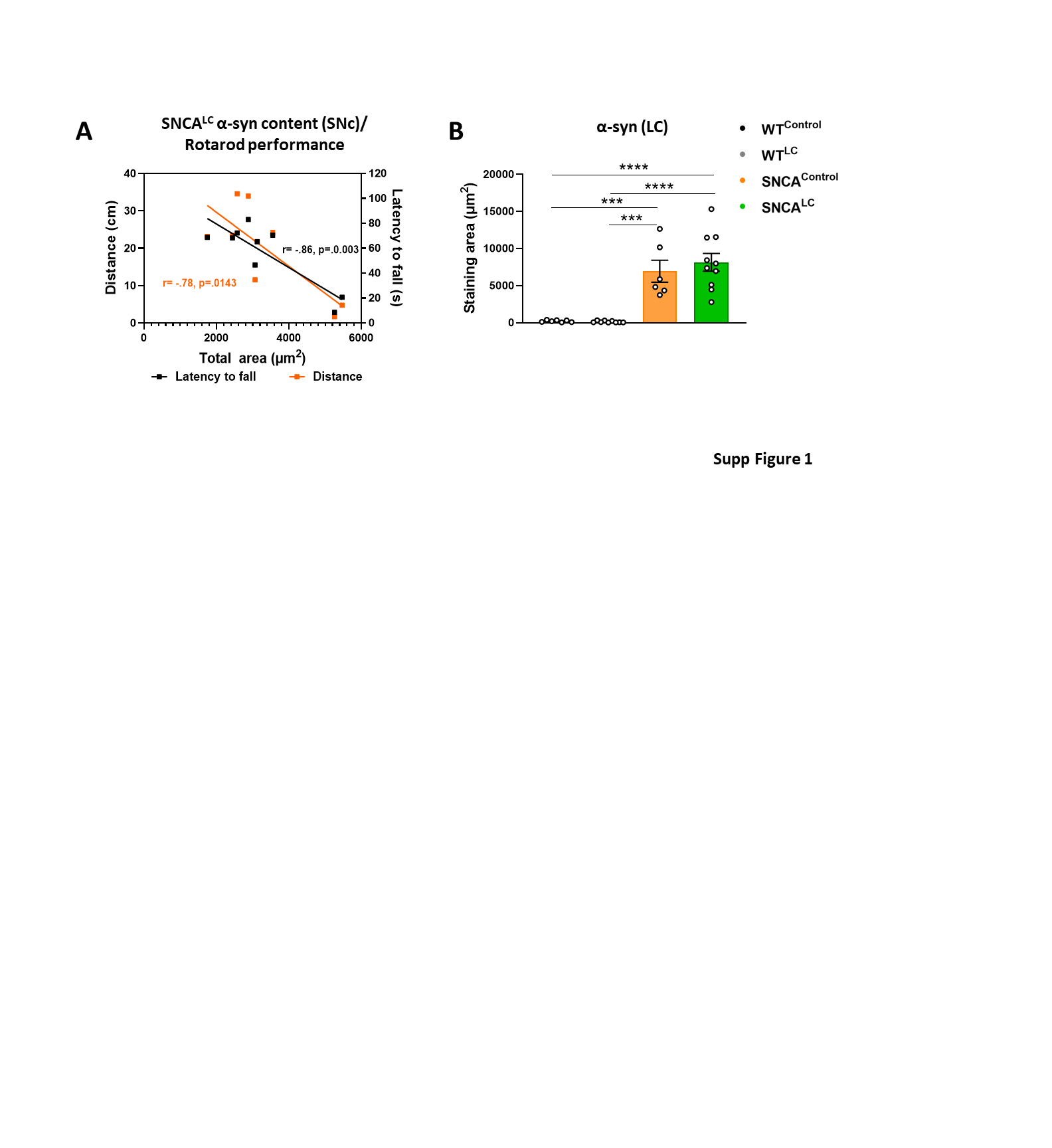


**Supplementary figure 2. Correlation analysis (SNc) and quantification of α-syn content in LC.** A- Distribution plot of Pearson correlation between α-syn aggregation in ventromedial part of SNc and distance crossed (orange) and latency to fall (black) in rotarod task in SNCA^LC^ mice. B- Quantification of total area of α-syn positive staining in the LC (n=6-10). ****p<0.00001, ***p<0.0001, all values denote means ± SEM.


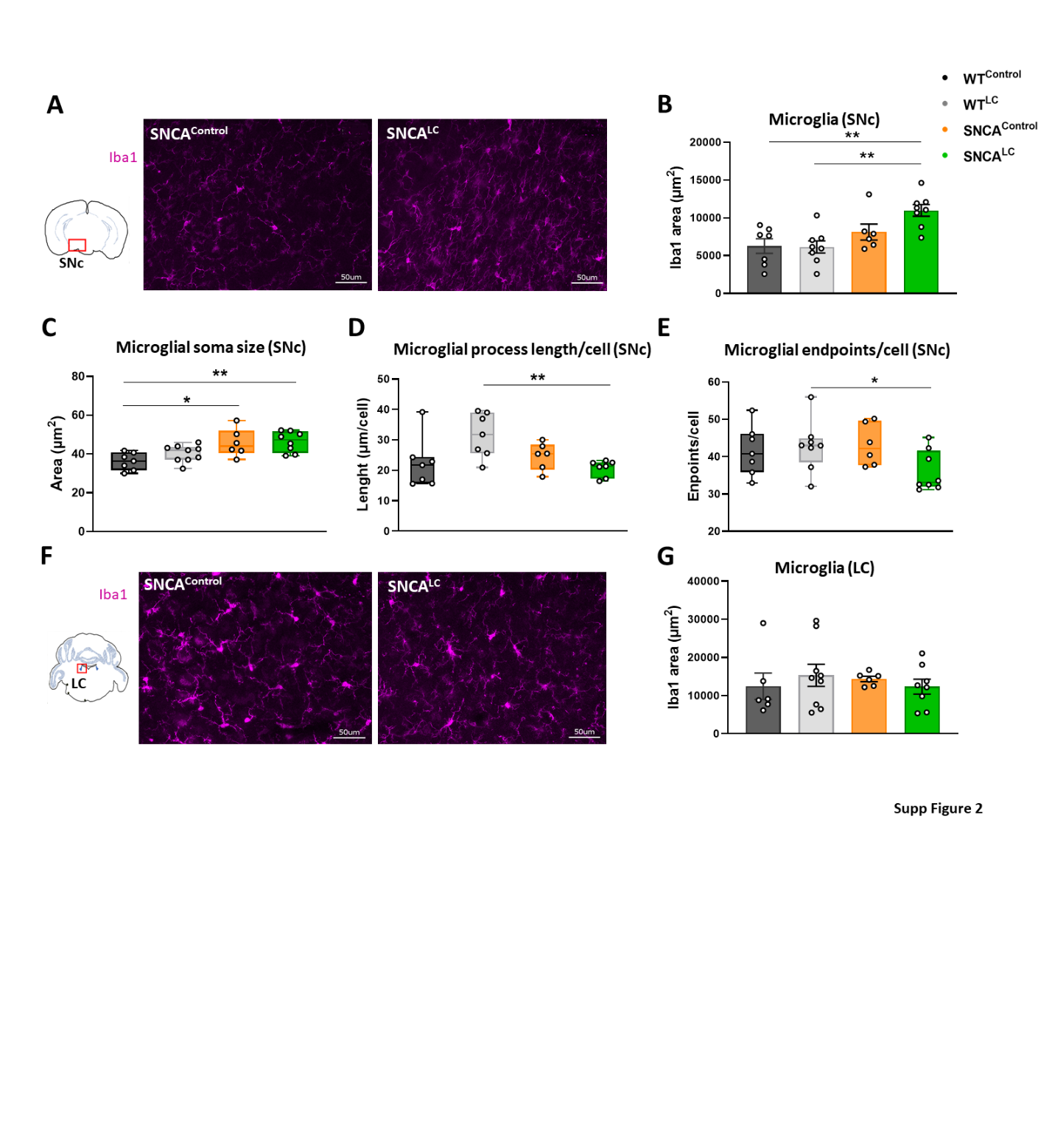


**Supplementary figure 3. Neuroinflammation upon α-synuclein aggregation is independent of LC-NE action.** A- Representative immunofluorescent images of microglial Iba1 staining in SNc of SNCA^Control^ and SNCA^LC^ mice, Iba1 (magenta), scale bar 50µm. B- Quantification of microglia presence by iba1 staining in SNc (n=6-8). C, D, E- Analysis of microglia soma size, endpoints per cell and process length per cell within SNc (n=6-8). F- Representative immunofluorescent images of Iba1 staining in LC of SNCA^Control^ and SNCA^LC^ mice, Iba1 (magenta), scale bar 50µm. G- Quantification of microglia presence by iba1 staining in LC (n=6-9). **p<0.001, *p<0.05, all values denote means ± SEM.


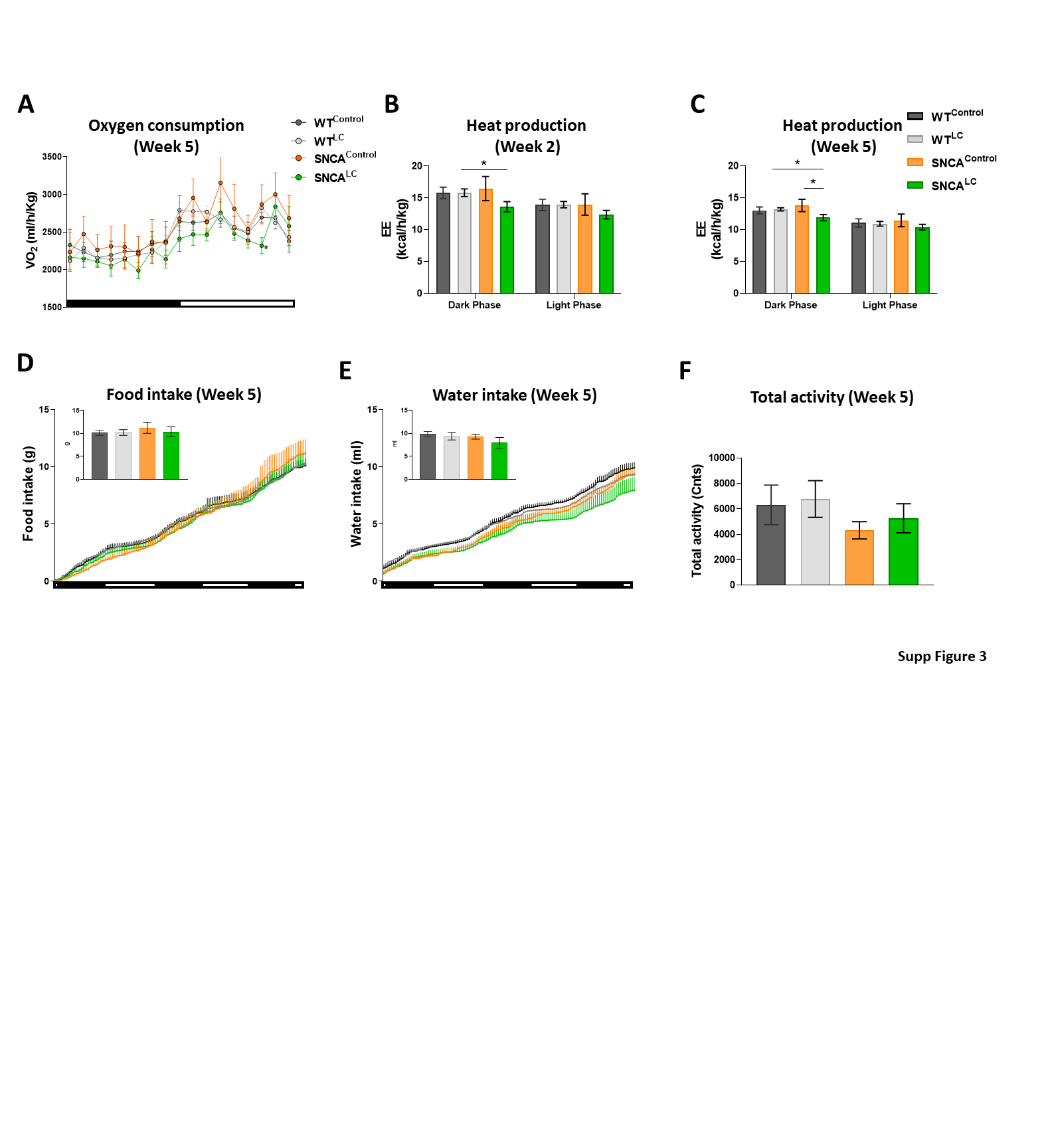


**Supplementary figure 4. Chronic stimulation of LC-NE neurons induces metabolic decline in α-syn overexpressing animals.** A - Oxygen consumption over the course of 1 day on week of chronic CNO treatment. B,C- Average heat production during light/dark phase obtained by indirect calorimetry on week 2 and week 5 of chronic CNO treatment. D- Cumulative food intake obtained by indirect calorimetry on week 5 of chronic CNO treatment, upper left panel on figure D: food intake on last time point, (n=6-9). E- Cumulative water intake obtained by indirect calorimetry on week 5 of chronic CNO treatment, upper left panel on figure E: water intake on last time point, (n=6-9). F- Total activity over the course of 4 days obtained by TSE Phenomater metabolic cage sensors on week 5 of chronic CNO treatment. *p<0.05, all values denote means ± SEM.

**Supplementary figure 5. Dose-dependent effect of NE on neuronal survival and growth.** A- Primary dopaminergic neurons survival in DMEM-F12 supplemented with Glutamax, B27 with antioxidants. B- Primary dopaminergic neurons culture in DMEM-F12 supplemented with Glutamax, B27 without antioxidants and representation of neurite analysis showing examples of roots, branch points, and total extremities. C- NE dose-dependent increase in TH neuron survival and neurite sprouting in medium without antioxidants. ****p<0.00001, ***p<0.0001, *p<0.05, all values denote means ± SEM.


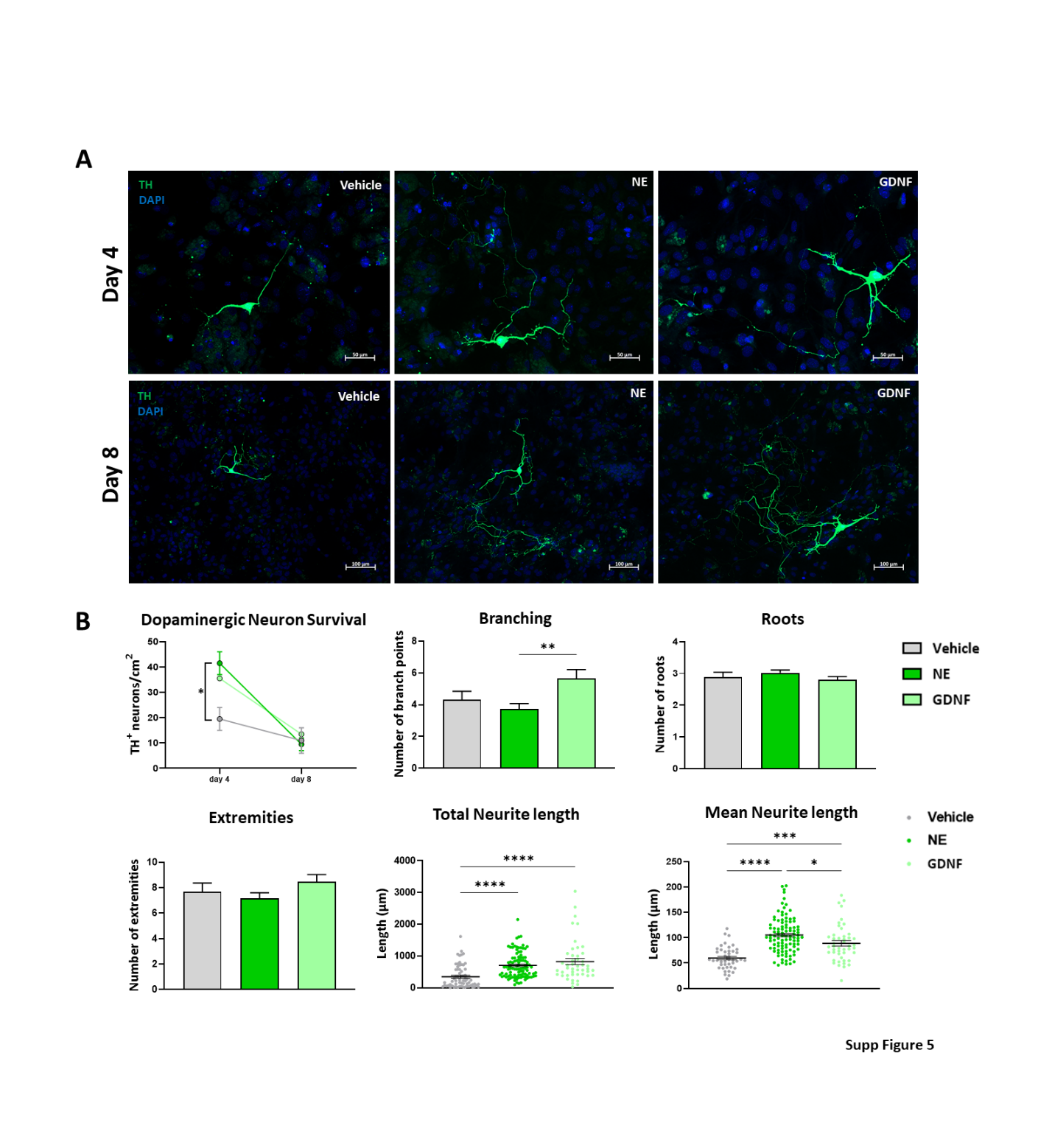
**Supplementary figure 6.** **Effect of different types of culture medium on neuronal survival and growth**. A - Representative images of TH immunofluorescent staining on day 4 and day 8, respectively, of primary dopaminergic neurons in medium without antioxidants, supplemented with vehicle, NE (1μM) or GDNF (100 ng/mL), TH (green), DAPI (blue), scale bar 50 μm and 100 μm, respectively. B- TH neurons survival at day 4 and day 8, neurite growth analysis at day 4. ****p<0.00001, ***p<0.0001, **p<0.001, *p<0.05, all values denote means ± SEM.


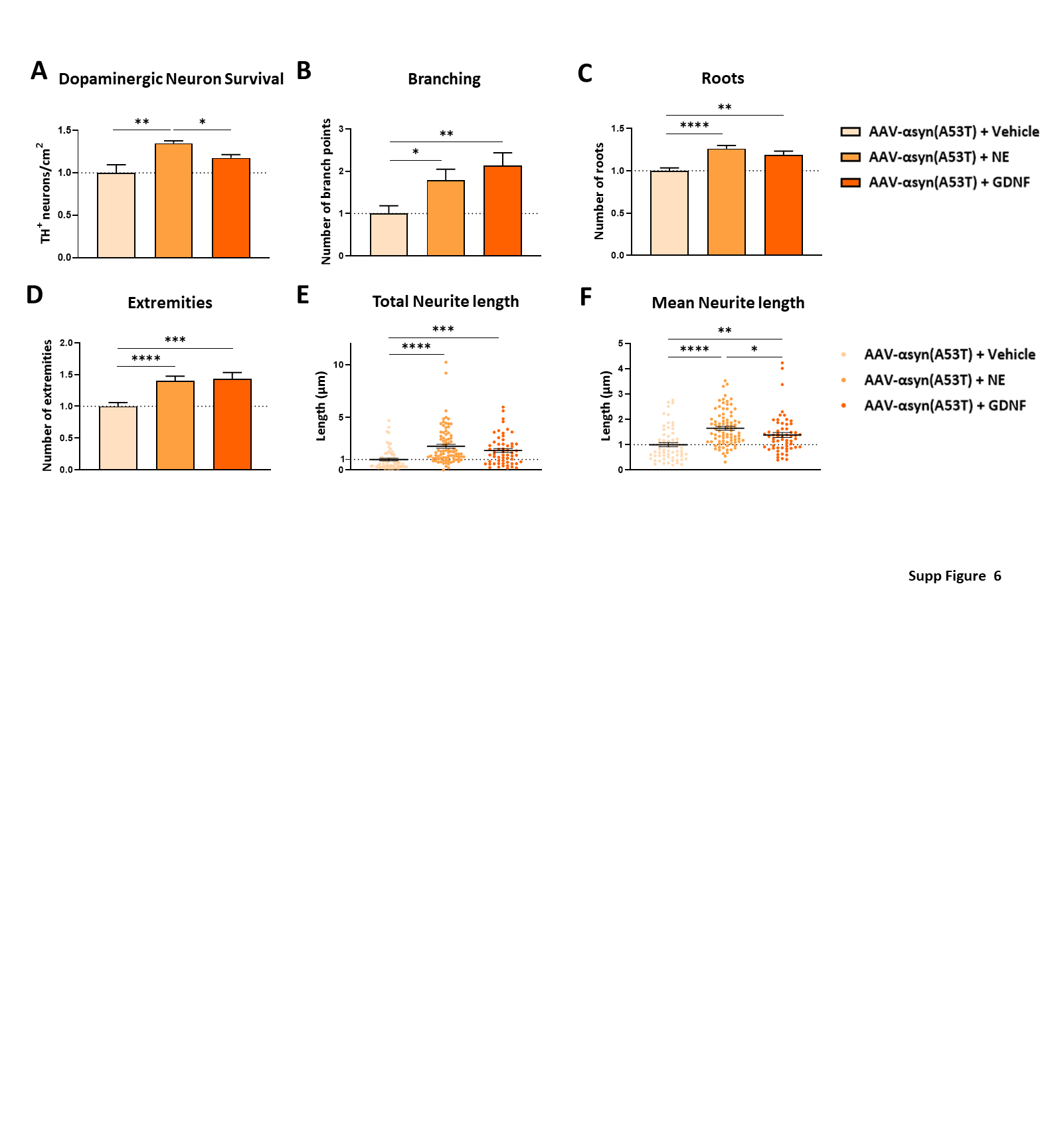
**Supplementary figure 7. Comparison of neuroprotective effect of NE and GDNF in AAV-α-synuclein transduced primary DA cell culture.** A- Relative fold change of dopaminergic neurons survival and B-F neurite growth analysis at day 4. ****p<0.00001, ***p<0.0001, **p<0.001, *p<0.05, all values denote means ± SEM.
